# Diatom-inspired multiscale mineralization of patterned protein–polysaccharide complex structures

**DOI:** 10.1093/nsr/nwaa191

**Published:** 2020-08-29

**Authors:** Ke Li, Yingfeng Li, Xinyu Wang, Mengkui Cui, Bolin An, Jiahua Pu, Jintao Liu, Boyang Zhang, Guijun Ma, Chao Zhong

**Affiliations:** School of Physical Science and Technology, ShanghaiTech University, Shanghai 201210, China; School of Physical Science and Technology, ShanghaiTech University, Shanghai 201210, China; School of Physical Science and Technology, ShanghaiTech University, Shanghai 201210, China; School of Physical Science and Technology, ShanghaiTech University, Shanghai 201210, China; School of Physical Science and Technology, ShanghaiTech University, Shanghai 201210, China; School of Physical Science and Technology, ShanghaiTech University, Shanghai 201210, China; School of Physical Science and Technology, ShanghaiTech University, Shanghai 201210, China; School of Physical Science and Technology, ShanghaiTech University, Shanghai 201210, China; School of Physical Science and Technology, ShanghaiTech University, Shanghai 201210, China; School of Physical Science and Technology, ShanghaiTech University, Shanghai 201210, China; Center for Materials Synthetic Biology, Shenzhen Institute of Synthetic Biology, Shenzhen Institutes of Advanced Technology, Chinese Academy of Sciences, Shenzhen 518055, China; CAS Key Laboratory of Quantitative Engineering Biology, Shenzhen Institute of Synthetic Biology, Shenzhen Institutes of Advanced Technology, Chinese Academy of Sciences, Shenzhen 518055, China

**Keywords:** biomimetic mineralization, patterned porous structure, genetic engineering, amyloid protein, artificial photosynthesis

## Abstract

Marine diatoms construct their hierarchically ordered, three-dimensional (3D) external structures called frustules through precise biomineralization processes. Recapitulating the remarkable architectures and functions of diatom frustules in artificial materials is a major challenge that has important technological implications for hierarchically ordered composites. Here, we report the construction of highly ordered, mineralized composites based on fabrication of complex self-supporting porous structures—made of genetically engineered amyloid fusion proteins and the natural polysaccharide chitin—and performing *in situ* multiscale protein-mediated mineralization with diverse inorganic materials, including SiO_2_, TiO_2_ and Ga_2_O_3_. Subsequently, using sugar cubes as templates, we demonstrate that 3D fabricated porous structures can become colonized by engineered bacteria and can be functionalized with highly photoreactive minerals, thereby enabling co-localization of the photocatalytic units with a bacteria-based hydrogenase reaction for a successful semi-solid artificial photosynthesis system for hydrogen evolution. Our study thus highlights the power of coupling genetically engineered proteins and polysaccharides with biofabrication techniques to generate hierarchically organized mineralized porous structures inspired by nature.

## Teaser

Inspired by the exquisite diatom frustule, genetically engineered amyloid proteins bearing mineralization-promoting domains are combined with chitin to fabricate and then mineralize a variety of hierarchically ordered porous structures.

## INTRODUCTION

Biological organisms such as diatoms, sponges and radiolarians are able to make hierarchically organized, mineralized structures across multiple length scales [1–3]. One particularly interesting example is diatoms—unicellular, eukaryotic algae—which are well known to produce a wide variety of hierarchically ordered porous silica structures in a genetically controlled manner [1–4]. The diatom frustule (i.e. the hard and porous external layer of diatoms) is multifunctional: it serves as a protective barrier between the cytoplasm and the exterior environment, and it also provides mechanical strength to predation [3,4]. Furthermore, the micro-scale, periodic structure of the frustule possesses interesting light-directing properties, which diatoms are known to exploit to enhance their photosynthetic capacity [5–7]. Accordingly, diatoms have long served as a fruitful source of inspiration for fabrication of novel bio-inspired hierarchically porous materials.

Over several decades, numerous strategies have been used for *in vitro* generation of nanostructured silica-based material structures, including, for example, use of organic molecules (such as surfactants, polymers and organo-gelators) to guide silica mineral formation [8–13]. Most organic templates used in these efforts have been based on long-chain cationic polymers, mainly polyamines, to mimic the positively charged sequences of the silaffin proteins known to orchestrate diatom frustule biogenesis [14–19]. These organic templates can control the size and morphology of the silica particles that form; however, to date, these strategies have yielded relatively simple shapes such as rods, spheres or hexagonal platelets [14,20–23]. Other research efforts have focused on application of various three-dimensional assemblies of organic matrices (cellulose, collagen and virus) for structured aggregation of silica particles to form higher-order silica structures [14,15,24–30]. These *in vitro* scaffolds have led to controlled deposition and organization of silica and some metal oxide minerals [31–33]. Additionally, diatom frustule-inspired scaffolds such as DNA origami architectures and micro-patterned functional silk structures have been explored to produce spatially ordered, nano- or micro-sized mineralized composites [34,35]. Despite important advances in biomimetic mineralization research, the development of mineralized structures recapitulating the hierarchically ordered porous structures of diatoms across multiple length scales remains elusive.

**Figure 1.**
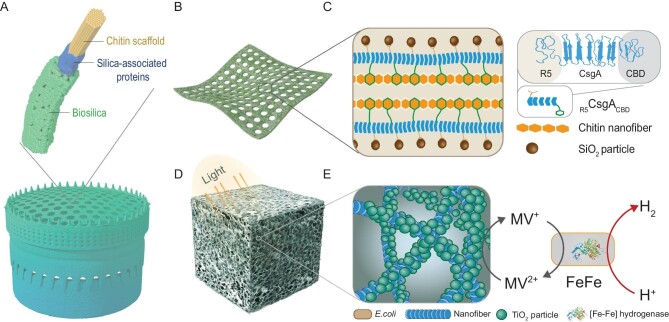
Bio-inspired construction of self-supporting porous structures using engineered multifunction amyloid fusion proteins as scaffolds for *in situ* mineralization. (A) Schematic of a typical diatom ordered porous structure comprising a chitin scaffold, silica-associated proteins and biosilica. (B and C) Schematic of the components of self-supporting patterned porous sheets (PPS) obtained by engineering strong amyloid/chitin interactions within the nanofiber matrix. (D and E) Schematic illustration of an artificial photosynthesis system for H_2_ evolution based on 3D mineralized _R5_CsgA_CBD_/chitin cubes.

Here, aiming to recapitulate the hierarchically porous mineral structures and functional properties of diatom frustules (Fig. 1A), we developed a biomimetic mineralization strategy in which complex porous structures made of genetically engineered multifunctional amyloid fusion proteins and the polysaccharide chitin are used as scaffolds for *in situ* mineralization of both SiO_2_ and metal oxides across multiple length scales. Our design was inspired by biogenesis of mineralized frustule structures. First, a three-domain fusion protein was engineered to comprise the CsgA protein from the well-known curli amyloid system of *Escherichia coli* (*E. coli*) [36,37], a chitin binding domain (CBD) from *Bacillus circulans* [38] and the R5 silica-nucleation peptide from *Clavulinopsis fusiformis* [24,39–41]. This design aimed to mimic the molecular interactions intrinsic to natural diatom systems in which chitin-based networks serve as a framework

for silica mineralization during frustule formation, while soluble biomolecules such as silaffins are then used as silica-nucleating proteins to promote mineralization within the chitin matrix [39–42]. Second, two types of porous structures were produced to morphologically resemble the porous features of the diatom frustule, including a highly ordered 2D porous sheet fabricated via replica molding and a 3D irregular porous cube created using sugar cube as a template (Fig. 1B and D).

Using a biomimetic mineralization strategy, the 2D patterned and 3D irregular porous self-supporting protein/chitin composite structures can further template the formation of complex mineral architectures across multiple length scales (from nm to cm) (Fig. 1C and E). Extending the application of these multiscale mineralized structures, we demonstrate a hybrid artificial photosynthesis system based on a sugar-cube templated, 3D, biocompatible structure mineralized with photoreactive TiO_2_ and colonized with hydrogenase-expressing bacteria for successful hydrogen production (Fig. 1E).

## RESULTS

### Bio-inspired construction of silicified self-supporting porous _R5_CsgA_CBD_/chitin sheets

To mimic the multiscale mineralization of diatom frustules, we started by rationally designing self-assembling nanofiber networks capable of both promoting local silica nucleation and mineralization at the molecular level and recapturing protein–polysaccharide interactions that occur in diatom frustules. Specifically, we initially constructed and tested mineralization-promoting CsgA fusion proteins by genetically appending a SiO_2_-nucleation peptide (R5) at the N-terminus of CsgA (_R5_CsgA), a bacterial structural amyloid protein of *E. coli* biofilms that is amenable for genetic modification to endow functional properties without disrupting its nanofiber self-assembly capability [37,43]. We confirmed that the _R5_CsgA fusion proteins could self-assemble into amyloid nanofibers (Supplementary Fig. 1) and found that they could indeed serve as a template for promoting silica formation (Supplementary Fig. 2). To engineer protein–polysaccharide molecular interactions, we designed CsgA fusion proteins containing CBD at C-terminus (CsgA_CBD_). For context, the CsgA_CBD_ fusion proteins had previously been demonstrated by our research group to enable strong and specific protein–chitin molecular interactions, resulting in networked chitin–amyloid nanofiber soft hybrid materials [37].

**Figure 2.**
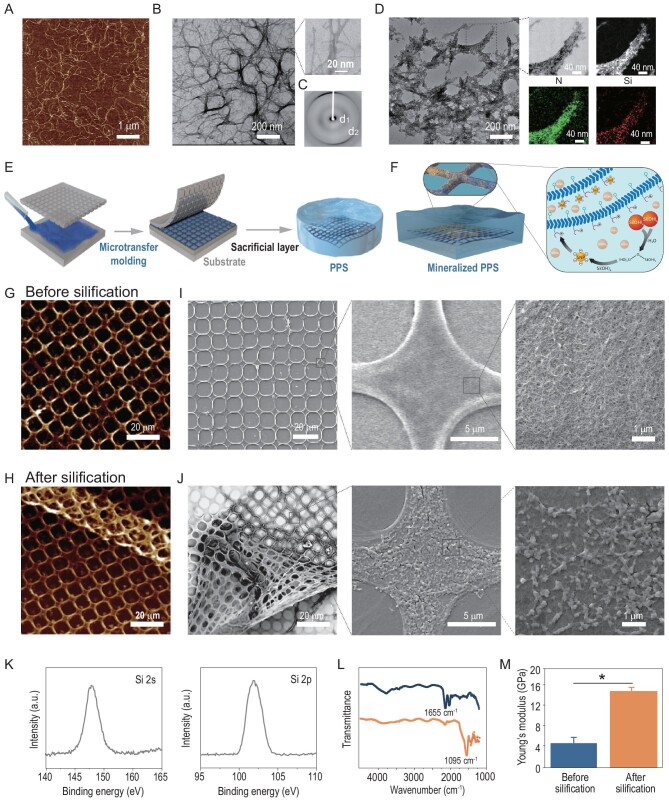
Morphologies, structural characterization and mechanical properties of rationally designed self-assembling _R5_CsgA_CBD_ nanofibers, self-supporting PPS and their corresponding mineralized architectures. (A–C) Morphological and structural characterization of the native amyloid nanofibers which self-assemble from the _R5_CsgA_CBD_ fusion proteins: (A) AFM image, (B) TEM images and (C) X-ray fiber diffraction pattern. (D) TEM images and STEM-EDS of the silicified _R5_CsgA_CBD_ nanofibers. (E) Schematic showing the bio-inspired construction of self-supporting PPS by coupling microtransfer molding with HFIP ink composed of dissolved _R5_CsgA_CBD_ monomers and squid pen polysaccharide β-chitin. (F) Schematic showing biomimetic mineralization of PPS in the presence of TEOS solution to produce silicified PPS. (G, H) AFM images of non-mineralized (G) and mineralized (H) complex PPS structures. (I, J) SEM images of non-mineralized (I) and mineralized (J) complex PPS structures. (K) XPS analysis of the silicified sheets on Al foil at the characteristic binding energies of 146 and 102 eV for electrons found in the 2s and 2p electron shells of the silicon atom, respectively. (L) ATR-FTIR spectroscopy of the silicified PPS; numbers indicate the peak wavenumbers (in 1095 cm^−1^) of the Si–O–Si antisymmetric stretching modes. (M) Young's modulus of _R5_CsgA_CBD_/chitin PPS before and after silicification. *P < 0.05, Student's *t* test. Note: the data were obtained by statistics from 256 × 256 spots in a 4 μm^2^ square per sample.

Our final functional protein module was therefore settled on engineered multidomain amyloid fusion proteins (_R5_CsgA_CBD_) in which the R5 peptide and CBD were genetically fused at the N- and C-termini of CsgA, respectively (Supplementary Figs 3 and 4). Morphological characterization by atomic force microscopy (AFM) and transmission electronic microscopy (TEM) revealed that the _R5_CsgA_CBD_ fusion proteins in solution could self-assemble into nanofibers, with an average fiber diameter of 1.7 ± 0.4 nm and an average length of 1097.5 ± 181.0 nm (Fig. 2A, B and Supplementary Fig. 5). Further, structural characterization by X-ray fiber diffraction revealed that the nanofibers displayed a typical β diffraction pattern, with a meridional reflection (denoted as d_2_ in the diffraction pattern) at 4.8 Å, reflecting the spacing between β-strands within each layer of β-sheets and an equatorial reflection (denoted as d_1_ in the diffraction patterns) at 9.5 Å, corresponding to inter-sheet packing distances (Fig. 2C) [43].

We then tested the self-assembling nanofibers composed of _R5_CsgA_CBD_ fusion proteins as organic templates for silica formation. After 10-min incubation in a precursor solution of tetramethoxysilane (TEOS) at room temperature, spherical SiO_2_ particles were found to appear around the nanofibers, clearly suggesting that the nucleation process of SiO_2_ was guided by the nanofibers (Fig. 2D, left). In contrast, other CsgA fusion proteins that do not contain R5 domain seemed not to promote the deposition of SiO_2_ nanoparticles under the same silicification conditions (Supplementary Fig. 6). In addition, a series of chemical mapping analyses using an energy dispersive spectrometer (EDS) coupled with electron microscopy imaging confirmed the silica mineralization of the amyloid fibers comprising the _R5_CsgA_CBD_ fusion monomers (Fig. 2D, right).

We next designed self-supporting porous structures consisting of _R5_CsgA_CBD_/chitin complex components for multiscale silica mineralization, largely inspired from the porous structure of the diatom frustule. Using a simple microtransfer molding process, we fabricated complex self-supporting structures made of _R5_CsgA proteins and the polysaccharide chitin (Fig. 2E and G) [44]. Through a methanol-assisted *in situ* curing process, CsgA fusion proteins in the fabricated structures would regain their amyloid β-sheet structures with fibrous morphology [37]. Briefly, using an hexafluoroisopropanol (HFIP) solution containing both the engineered _R5_CsgA_CBD_ protein monomers and dissolved squid pen β-chitin molecules, we printed and cured structures that we term patterned porous sheets (PPS) (Fig. 2E). Upon methanol vapor exposure, the protein monomers in the fabricated complex structures indeed reassembled into their characteristic nanofiber structures (Fig. 2G and I). In addition, the PPS composed of chitin and _R5_CsgA_CBD_ fusion proteins (length, ∼1 cm; width, ∼1 cm; thickness, 412 ± 15 nm and pore diameter, ∼10 μm) exhibited impressive durability and stability after exposure to aqueous solution, in sharp contrast to the deterioration we observed for incomplete PPS structures built solely of _R5_CsgA_CBD_ fusion proteins (i.e. lacking the polysaccharide chitin) (Supplementary Fig. 7). When these patterned porous sheets were exposed to a mineralization precursor solution of tetramethoxysilane for *in situ* mineralization (Fig. 2F), silica spheres appeared to form on the PPS surface, as revealed by AFM and scanning electron microscopy (SEM) (Fig. 2H and J). In contrast, deposition of silica spheres was not clearly found on the pure chitin PPS sheets, highlighting the role of fusion proteins in promoting silica formation (Supplementary Fig. 8). In addition, silica deposition was found to occur preferably at neutral pH (7.5), rather than at basic (10.5) or acidic (4.5) pH value (Supplementary Fig. 9), in agreement with phenomena reported in previously published works [24,45]. Examination of the mineralized structures by X-ray photoelectron spectroscopy (XPS) confirmed successful PPS silicification: these mineralized PPS exhibited characteristic binding energies of 146 eV and 102 eV for electrons found in the 2s and 2p electron shells of the silicon atom, respectively (Fig. 2K). Further characterization by Fourier Transform Infrared Spectroscopy (FTIR) revealed peak wavenumbers at 1095 cm^−1^, indicating that Si–O–Si antisymmetric stretching was occurring in the sheets (Fig. 2L). Highlighting the impressive mechanical properties of PPS after mineralization, we also used peak force quantitative nanomechanical (PK-QNM) AFM to measure the Young's modulus of non-mineralized and mineralized sheets and found that mineralization substantially increased the Young's modulus of the material by around 200% (from 4.84 ± 0.46 GPa to 14.16 ± 0.58 GPa) (Fig. 2M).

Collectively, by integrating rationally designed mineralization-promoting amyloid proteins with a microtransfer molding process, we precisely fabricated ordered porous structures that can be mineralized *in situ* across multiple scales, thereby resulting in hierarchically ordered mineralized structures resembling the exquisite frustules of diatoms [44]. These results implied that the _R5_CsgA_CBD_ fusion proteins can serve as self-assembling nanofibers that promote local silica mineralization. The SiO_2_ mineral substantially enhances the mechanical properties of the free-standing PPS structures, thus potentially broadening a wide range of applications including serving as protective shelters for biomolecules and mechanical strengthening building materials [34]. For example, immobilization of enzymes in mechanically stable and chemically inert silica matrices would provide the embedded enzymes with better environmental tolerance and extended life-time storage, likely extending application of these biomolecules even under non-physiological conditions [25,46].

### Biomimetic mineralization of _R5_CsgA_CBD_/chitin materials with TiO_2_

Recalling the known ability of the R5 peptide to mediate the mineralization of TiO_2_ [31], we next investigated whether the engineered _R5_CsgA_CBD_ amyloid nanofibers could nucleate metal oxide to generate mineralized nanostructures. The use of biomimetic mineralization approaches to produce porous metal oxide-based composites at room temperature is particularly attractive, given that metal oxide-based materials are known to be useful for a wide variety of applications in photocatalysis and photovoltaics [32].

**Figure 3.**
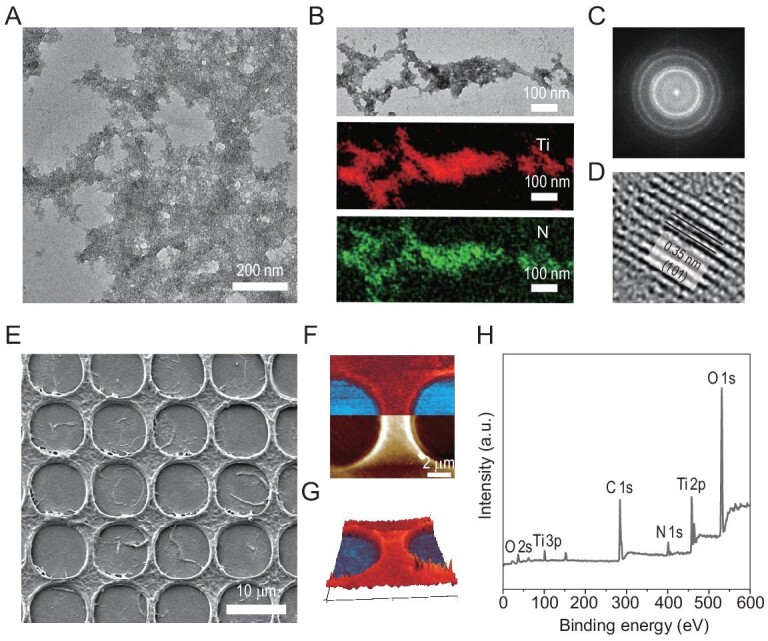
Biomimetic mineralization of TiO_2_ with self-assembling _R5_CsgA_CBD_ nanofiber networks and self-supporting PPS. (A–C) Morphological, elemental and structural analysis of the mineralized _R5_CsgA_CBD_ nanofibers: (A) TEM image, (B) TEM-EDS images and (C) diffraction pattern. Note the diffraction ring representative of a typical anatase phase for TiO_2_ mineral. (D) HR-TEM analysis of a microsphere fragment showing the (101) lattice fringes of the crystal. (E–H) Morphological and structural analysis of the mineralized PPS: (E) SEM image, (F) AFM topography (bottom) and IR absorption (top) images showing where mineralized TiO_2_ (using Ti-O stretch absorption at 750 cm^−1^) were localized on the sheets. (G) The 3D overlaid image combining topography and IR absorption showing the homogeneous and topographic distribution of TiO_2_ on the patterned sheets. Note: red and blue colors indicate strong and weak Ti-O stretch absorption, respectively. (H) XPS survey spectra of TiO_2_ mineralized _R5_CsgA_CBD_ PPS showing the chemical identity.

We started to assess whether the engineered _R5_CsgA_CBD_ amyloid nanofibers could template the nucleation and growth of TiO_2_. To such ends, we added nanofibers to buffered aqueous solutions containing Ti (IV) bis-(ammonium lactato)-dihydroxide (TiBALDH) to trigger TiO_2_ mineralization. We found that the solutions became turbid when the _R5_CsgA_CBD_ nanofibers were added, implying their potential as self-assembling scaffolds to promote TiO_2_ mineralization. We confirmed the successful mineralization of the R5-interacting TiO_2_ using multiple analyses including EDS, selected area electron diffraction (SAED) and lattice fringe high-resolution transmission electron microscopy (HR-TEM). TEM and corresponding EDS analysis indicated the presence of Ti on the surface of the _R5_CsgA_CBD_ nanofibers exposed to the mineralization precursor solution and the presence of N (from the proteinaceous components) (Fig. 3A and B). Further, the SAED pattern obtained from the coatings close to the nanofiber surface revealed rings with spots having d-spacing of 3.5, 2.4 and 2.3 Å, corresponding to the lattice plane (101), (004) and (103), respectively (anatase phase, JCPDS 84–1286) (Fig. 3C). The relative intensities of the diffraction patterns matched the three most intense values for nanocrystalline TiO_2_ (anatase phase, JCPDS 84–1286). Further, the lattice fringe spacings were consistent with the (101) plane spacing of anatase (anatase phase, JCPDS 84–1286) (Fig. 3D).

Having demonstrated successful mineralization at fibril level, we next assessed TiO_2_ mineralization using the self-supporting porous sheets (Fig. 3E). After mineralization, we used nanoscale-infrared spectroscopy (nano-IR) at a wavenumber of 750 cm^−1^ to reveal the presence of TiO_2_ on the mineralized sample, and indeed an overlay of the reconstituted topography image (3D) with a color code representing the absorption intensity at 750 cm^−1^ clearly indicated the homogeneous distribution of TiO_2_ on the sheets (Fig. 3F and G). Moreover, corresponding chemical mapping analyses based on EDS further indicated the successful TiO_2_ mineralization (Supplementary Fig. 10). In addition, XPS spectral analysis revealed that the mineralized sheets had distinct signals for oxygen (O 1s and 2s), carbon (C 1s), nitrogen (N 1s) and titanium (Ti 2p and 3p) (Fig. 3H), again supporting successful TiO_2_ mineralization of the PPS. We next measured the Young's modulus to assess the effects of TiO_2_ mineralization on the mechanical properties of PPS based on PK-QNM AFM methodology: the mineral indeed increased the elasticity of the PPS (from 4.84 ± 0.46 GPa to 7.21 ± 0.51 GPa) (Supplementary Fig. 11). Notably, using a similar biomimetic mineralization approach, Ga_2_O_3_, an ultra-wide bandgap oxide semiconductor, could also be mineralized on both nanofibers and porous _R5_CsgA_CBD_/chitin sheets (Supplementary Figs 12–14). Collectively, these results establish that porous _R5_CsgA_CBD_/chitin sheets can serve as scaffolds for templating the *in situ* growth of metal oxides to produce mineralized porous sheets over multiple length scales.

### Fabrication of self-supporting and high surface area mineralized porous structures

The hierarchical porous structures of diatoms endow them with adaptive functions [5–7], providing inspiration for reconstructing artificial hierarchical porous materials for numerous applications. Previous studies have demonstrated that hierarchically structured porous bioceramic-silk composites could enhance cell attachment, proliferation and gene expression, and porous silica-based structures could facilitate enzyme immobilization, while porous organic polymers serve as a promising platform for designing heterogeneous catalysts [47–49]. Motivated by the applications of such diatom-inspired structures, we turned to construct porous mineralized _R5_CsgA_CBD_/chitin composites with high-surface areas and photo-reactivity for proof-of-concept demonstration of a solar-driven hydrogen evolution system.

**Figure 4.**
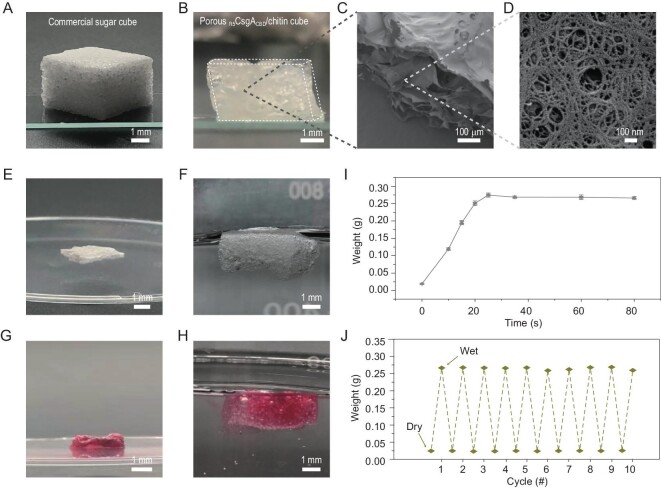
Morphological characterization and reversible dehydration/hydration behaviors of the fabricated porous _R5_CsgA_CBD_/chitin cube. (A, B) Digital photographs of the commercial sugar cube (A) and the fabricated porous _R5_CsgA_CBD_/chitin cube in initial hydration state (B). (C) SEM image showing the internal porous structures of the fabricated _R5_CsgA_CBD/_chitin cube. (D) SEM image of a zoomed-in area from (C), revealing that the cube is composed of nanofibers. (E–H) Digital camera snapshots of the same porous _R5_CsgA_CBD_/chitin cube switching between dehydration (dry) state and hydration state in different solutions: a dry sample (E) swells and floats at the surface of aqueous solution, resulting in swollen sponge (F); the dehydrated sample (G) from (H) re-swells into original sponge-like shape in Congo red solution. (I) Weight changes of the porous _R5_CsgA_CBD_/chitin cube as a function of swelling time in aqueous solution. Data are presented as mean ± SD (*n* = 3 repeats). (J) Weights of the same cube in hydration and dehydration state after multiple cycles of hydration/dehydration, illustrating the reversible hydration/dehydration behavior of the porous _R5_CsgA_CBD_/chitin cube. Note: To obtain completely dry samples, filter papers and constant N_2_ blowing were applied to remove the trapped water in the hydrated samples.

To such ends, we started by preparing a 3D porous _R5_CsgA_CBD_-chitin complex scaffold for TiO_2_ mineralization. We first applied HFIP ink containing both _R5_CsgA_CBD_ monomers and chitin, along with a porous sugar cube as a bulk template, to fabricate 3D complex self-supporting structures (Fig. 4A). After 12-h immersion in HFIP ink, the sugar cube was cured under methanol vapor to trigger the reassembly of amyloid proteins into nanofibers throughout the whole matrix of the cube. After dissolving the sugar cube template in aqueous solution, a self-supporting 3D amyloid-chitin complex structure was obtained. The successful replication of the sugar cube shape as a sponge-like structure was evident in normal photographs (Fig. 4B), and the porous surfaces of the scaffold were found to comprise a large amount of nanofiber structures under SEM (Fig. 4C and D). Notably, the air-dried 3D porous structures could spontaneously restore their shapes in their hydration state after immersion in different solutions (e.g. distilled water and Congo red solution) (Fig. 4E–H and Supplemental Fig. 15), and rapidly reached equilibrium swelling ratio in solution within 25 s (Fig. 4I). Additionally, these structures exhibited reversible hydration/dehydration behaviors with nearly identical swelling/de-swelling ratios even after multiple cycles (Fig. 4J), thus allowing for recyclable loading and release of specific reagents in a controlled manner.

With their rapid swelling feature and reversible swelling behavior, we hypothesize that the fabricated porous cubes would first serve as sponge-like materials to homogeneously absorb a mineralization precursor solution for TiO_2_ mineralization, and then harbor a mixed reaction solution containing bacterial cells for artificial photosynthesis. Ideally, in the final constructed artificial photosynthesis system, mineralized TiO_2_ nanoparticles would act as the light-antennae component converting photons into electrons, and an engineered *E. coli* strain harboring a hydrogenase gene cluster, upon receiving electrons transported by methyl viologen (MV), could catalyze continuous hydrogen evolution when the system was illuminated [50].

**Figure 5.**
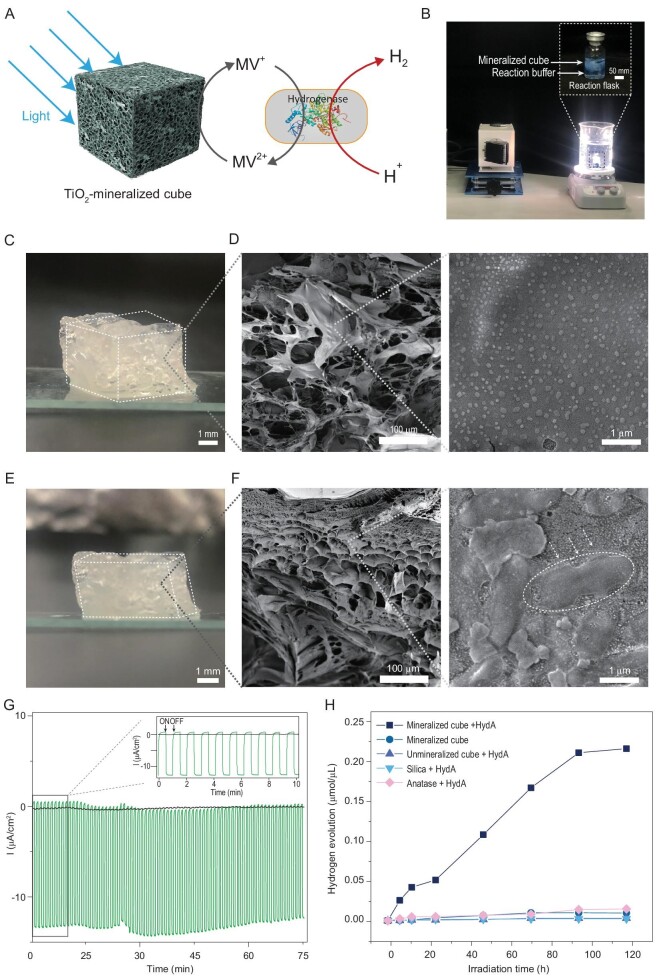
Bio-inspired construction of an artificial photosynthesis system for hydrogen evolution by coupling high surface area, self-supporting, 3-D porous mineralized cubes with colonized engineered *E. coli* cells. (A) Schematic illustration of an artificial photosynthesis system for H_2_ evolution based on the mineralized _R5_CsgA_CBD_/chitin cubes, in which mineralized TiO_2_ nanoparticles act as light-antennae for converting photons into electrons and engineered *E. coli* cells harboring a hydrogenase gene cluster are in close proximity to the TiO_2_ structures, enabling constant hydrogen evolution upon receiving electrons delivered by methyl violet (MV). (B) Photograph image of hydrogen evolution equipment for the artificial photosynthesis systems based on mineralized porous cube structures. (C, D) Morphologies of non-mineralized and mineralized porous _R5_CsgA_CBD_/chitin cubes before loading with engineered *E. coli* cells: (C) photograph images of mineralized porous _R5_CsgA_CBD_/chitin cubes; (D) SEM images of mineralized porous _R5_CsgA_CBD_/chitin cubes. (E, F) Morphologies of the mineralized porous _R5_CsgA_CBD_/chitin cubes after incubation with engineered *E. coli* cells: (E) photographic and (F) SEM images. (G) Transient photocurrents generated by mineralized TiO_2 R5_CsgA_CBD_/chitin porous cubes. The green curve represents the photocurrent of the mineralized _R5_CsgA_CBD_/chitin cube structure deposited on FTO glass, when illuminated (on) or shielded (off) from visible light. The black curve represents the photocurrent of non-mineralized _R5_CsgA_CBD_-chitin materials on FTO glass. (H) H_2_ evolution over time catalyzed with different hybrid systems: the mineralized cube reaction system (navy blue curve), the system utilizing excessive amount of free TiO_2_ nanoparticles (pink curve), the cubic system lacking engineered strain (green curve), the cubic system containing the engineered strain but lacking mineralized TiO_2_ (indigo curve) and the SiO_2_ mineralized scaffolds containing engineered strain (light green curve). Note: The light source used here was a 300 W Xenon lamp (CEL-HXF300, CEAULIGHT) with illumination intensity of 20.07 mW/cm^2^. A three-electrode system was used for photocurrent testing. A platinum wire and an Ag/AgCl were used as counter and reference electrodes, respectively. The applied potential was 0 V, and the electrolyte solution was 0.5 M Na_2_SO_4_. The solution was purged with continuous N_2_ bubbles for 30 min to get rid of oxygen in the electrolyte cell. The experiments in G and H were replicated at least three times with identical results.

We next probed whether the fabricated porous 3D cubes could promote local TiO_2_ mineralization and whether the mineralized porous cubes could further absorb and harbor bacterial from aqueous solution (Fig. 5A and B). After 2-h incubation in a TiBALDH mineralization precursor solution, the 3D cube structures maintained their original shape (Fig. 5C). SEM and EDS analysis revealed that mineralized TiO_2_ nanoparticles could directly form on the fibers comprising the cube (Fig. 5D and Supplemental Fig. 16). The UV-Vis absorption spectra showing a sharp absorption edge at ∼385 nm representative of TiO_2_ bandgap excitation, further confirmed the successful mineralization of TiO_2_ in the porous cube (Supplementary Fig. 17).

Nanoindentation measurements showed that the Young's modulus of the TiO_2_-mineralized cube was obviously higher than that of the unmineralized cube (Supplementary Fig. 18), with values of 8.58 ± 1.63 GPa and 33.14 ± 6.58 GPa for the unmineralized and mineralized cube structures, respectively. In addition, after incubation of the mineralized cube in an aqueous solution containing the engineered *E. coli* strain, SEM analysis clearly indicated that the bacteria could distribute homogeneously inside the porous structure of the mineralized cube (Fig. 5E and F). These results therefore confirm that the 3D structures can serve as 3D scaffolds for biomimetic mineralization and for trapping bacterial cells.

### Artificial photosynthesis of the photoreactive mineralized porous structures

Biogenic hydrogen production coupling microbials with protective shelters is a well-known technique in the field. In particular, *cyanobacteria* (e.g. *Synechocystis* sp., *Microcystis aeruginosa, Phormidium valderianum*) have been used for hydrogen production via direct photolysis [51,52]. When encapsulated in a 1.5% agar matrix, the cyanobacteria *Oscillatoria* sp. showed that the rate and longevity of hydrogen production increased significantly compared to that of free cells [52]. The cyanobacteria *Synechocystis* sp. *PCC 6803* in silica matrices showed that hydrogen production of encapsulated cells was higher than that of cells in suspension [53,54]. Despite these advances, hydrogen production efficiency using biological organisms has been limited by its low volumetric productivity. As such, there has been interest in semi-artificial photosynthesis coupling inorganic semiconductor catalysts with engineered cells for their high performance in terms of broad-spectrum absorption and light harvesting efficiency [55].

We next explored the use of mineralized TiO_2_ cubes in an artificial photosynthesis system for solar-driven hydrogen production. To such ends, we first measured the transient photocurrent of a mineralized amyloid cube by placing it onto a piece of FTO-conductive glass and dried the sample at 37°C overnight (Supplementary Fig. 19). The glass coated with the dried sample was then successfully used as an electrode: a transient photocurrent of 12.7 ± 1.1 μA/cm^2^ was detected when it was exposed to light and the value dropped to almost zero when illumination was terminated, thus highlighting its pronounced property of photoresponse (Fig. 5G). Moreover, we tested the voltammetry responses of a bare FTO substrate and of TiO_2_-mineralized cube deposited on the FTO electrode in 0.50 M aqueous Na_2_SO_4_ (pH = 6.3). No redox peak was observed for both mineralized TiO_2_ and bare FTO glass over a wide voltage range of −0.1 ∼ 1.4 V vs RHE, and the curves of their cyclic voltammetry are identical, implying the electrochemical stability of the TiO_2_-mineralized cube under aqueous conditions (Supplementary Fig. 20).

With this photocatalytic mineralized cube reaction system established, we next carried out H_2_ evolution in the aforementioned semi-solid artificial photosynthetic system comprising a bacterial strain that expresses a hydrogenase (and its required maturases), TEOA as the sacrificial agent and MV as the mediator. UV spectroscopy revealed peaks between 300–400 nm and 500–700 nm, confirming the MV^+·^ formation upon illumination (but not under dark conditions) (Supplementary Fig. 21) [56]. We first assessed how the reaction system performed in terms of hydrogen production under different conditions. As revealed, a final H_2_ concentration of 0.21 μmol/μL was obtained after 120 h reaction based on the mineralized cube reaction system (navy blue curve), which was substantially higher than that of the system using the excessive amount of free TiO_2_ nanoparticles (0.014 μmol/μL) (pink curve). In contrast, almost no H_2_ evolution was found for the cubic system lacking engineered strain (green curve), and for the cubic system containing the engineered strain but lacking mineralized TiO_2_ (indigo curve). Meanwhile, H_2_ production was not detected in the SiO_2_-mineralized scaffolds containing engineered strain (light green curve) (Fig. 5H). Based on the results, we determined the optimized conditions for our semi-artificial cubic system: the initial cell density applied is around 1 × 10^9^ colony forming units (cfu) and solution pH = 8. (Supplementary Fig. 22). Conceivably, the 3D porous cube provides an ideal confined space in which mineralized TiO_2_ and engineered bacterial are in good contact with each other, ensuring high-efficiency solar-driven hydrogen evolution. In addition, the results indicated that the hydrogen production efficiency of a biological enzymatic system could be substantially enhanced when combined with mineralized TiO_2_. We envision that our 3D porous mineralized material systems coupled with colonized engineered bacteria may serve as a viable alternative route for conducting various artificial photosynthetic reactions.

## CONCLUSION

Diatom frustules represent exquisite masterpiece examples of natural hierarchical composite materials, generating keen interest for exploring various diatom-inspired scaffolds for biomimetic mineralization. Despite important advances, it is still challenging to fully recapitulate the multiscale mineralization features and functions of natural diatom structures in current state-of-the-art biomimetic composites. In our view, the difficulties of biomimetic mineralization research to reconstruct the spectacular morphologies and their remarkable material properties of natural composite materials arise, at least in part, from the inability to precisely order the molecular recognition and interactions at the organic–inorganic interfaces (e.g. closely mimicking the polysaccharide–protein–mineral interactions) across multiple scales with existing bio-derived or bio-inspired scaffolds. Our self-supporting PPS and porous sponge-like architectures, made of genetically programmable amyloid proteins and polysaccharide chitin, for biomimetic multiscale mineralization have thus brought us a step closer to nature. As demonstrated, the produced 3D architectures can template the formation of diverse minerals across multiple length scales, and the resultant mineralized porous composites can be further used for performing solar-driven hydrogen evolution reactions, opening the door for exploiting porous mineralized structures for various artificial photosynthesis systems. Additionally, our studies will spur new interest in designing sophisticated 3D scaffolds for multiscale mineralization by rationally incorporating the hidden molecular recognitions among protein–polysaccharide–mineral interfaces of natural minerals through genetic engineering.

Moving forward, given the genetically engineerable aspect of our mineralization scaffolds, it should be quite straightforward to develop additional biomimetic mineralization fusion proteins by swapping the silica-nucleating R5 peptide for other peptides that are known to mediate the mineralization of inorganic nanomaterials, thereby substantially expanding the versatility and applications of this general biomimetic mineralization design strategy. For example, porous CoPt catalytic materials should be easily generated based on a cobalt-binding peptide Co1-P10 that can control the nucleation of CoPt nanoparticles, while porous hydroxyapatite (HA) composites can be fabricated by applying fusion proteins containing HA-promoting protein domains found in bone.

## STATISTICS

Data analysis was performed on Origin 2019b software and presented as mean ± SD (standard deviation), calculated based on at least three replicates. Statistical comparisons between two groups were based on Student's *t* test with two tailed distribution, and *P*-values less than 0.05 were considered to be statistically significant.

## Supplementary Material

nwaa191_Supplemental_File
