# Supplementary material for: Diatom-inspired multiscale mineralization of patterned protein–polysaccharide complex structures
Source: Natl Sci Rev. 2020 Aug 29;8(8):nwaa191. doi: 10.1093/nsr/nwaa191 (PMC8363331; doi:10.1093/nsr/nwaa191)
Supplement: nwaa191_Supplemental_File [file nwaa191_supplemental_file.docx]

# Supporting Information for:

**Diatom-inspired Multiscale Mineralization of Patterned Protein-polysaccharide Complex Structures**

*Ke Li^1 †^**, Yingfeng Li^1^**^†^, Xinyu Wang^1^, Mengkui Cui^1^, Bolin An^1^, Jiahua Pu^1^, Jintao Liu^1^, Boyang Zhang^1^, Guijun Ma^1^, Chao Zhong^1, 2, 3 *^*

^1^School of Physical Science and Technology, ShanghaiTech University, Shanghai 201210, China.

^2^Center for Materials Synthetic Biology, Shenzhen Institute of Synthetic Biology, Shenzhen Institute of Advanced Technology, Chinese Academy of Sciences, Shenzhen 518055, China

^3^CAS Key Laboratory of Quantitative Engineering Biology, Shenzhen Institute of Synthetic Biology, Shenzhen Institutes of Advanced Technology, Chinese Academy of Sciences, Shenzhen 518055, China

†These authors contributed equally to this work.

**^*^Corresponding author. Email:** [zhongchao@shanghaitech.edu.cn](mailto:zhongchao@shanghaitech.edu.cn)

## Supplementary Materials and Methods

**1. Chemicals used in this study**

**2. Sequences of proteins used in this study**

**3. Gene construction**

**4. Detailed protocols for SDS/PAGE and Western blotting SDS/PAGE**

**5. Preparation of functional CsgA fibers and functional CsgA fibers/chitin ink**

**6. Morphology characterization**

**7. Chemical composition characterization**

**8. Porous patterned sheet fabrication via a microtransfer molding process**

**9. Biomimetic mineralizaton**

**10. The sugar fab process for the mineralized cubes**

**11. Photocurrent detection from the mineralized cubes**

**12. Cyclic voltammetry measurements**

**13. Hydrogen evolution experiment**

**14. X-ray fiber diffraction of amyloid nanofibers**

**15.** **Quantitative assessment of Young’s modulus by PK-QNM**

**16. Nano-indentation for the Young’s modulus assessment**

**Supplementary Materials and Methods**

**1. Chemicals used in this study**

LB broth (MD bio), isopropyl-β-D-thiogalactopyranoside (IPTG, ultrapure, life science), carbenicillin disodium (carb, USP, Macklin), guanidine hydrochloride (GdnHCl, AR, Sinopharm), sodium chloride (NaCl, GR, Sinopharm), potassium dihydrogen phosphate (KH2PO4, AR, Sinopharm), potassium phosphate dibasic anhydrous (K_2_HPO_4_, AR, Macklin), imidazole (99%, Energy Chemical), sodium hydroxide (NaOH, AR, Sinopharm), hydrochloric acid (HCl, GR, General reagent), sulfuric acid (H_2_SO_4_, 98wt.%, Sinopharm), 1,1,1,3,3,3-hexafluoro-2-propanol (HFIP, SP, Aldrich), methanol (AR, Aldrich), polydimethylsiloxane (PDMS, SYLGARD184 Dow Corning), ethanol (SP, Macklin), acetone (SP, General Reagent), isopropyl alcohol (SP, Xilong Scientific), hexane ( SP, Aladdin), hydrogen peroxide solution (H_2_O_2_, 30wt.% in H_2_O, Aladdin), uranyl acetate (2 wt.%, Beijing Zhongjing Key Technology), Polyvinyl alcohol (PVA, 3000 Da, BR, Sangon Biotech), sodium dodecyl sulfate (SDS, Beyotime), and formic acid (GC, Macklin).

**2. Sequences of proteins used in this study**

*CsgA*

MGVVPQYGGGGNHGGGGNNSGPNSELNIYQYGGGNSALALQTDARNSDLTITQHGGGNGADVGQGSDDSSIDLTQRGFGNSATLDQWNGKNSEMTVKQFGGGNGAAVDQTASNSSVNVTQVGFGNNATAHQYHHHHHH

*_R5peptide_CsgA*

SSKKSGSYSGSKGSKRRILGGGGSGGGGSGVVPQYGGGGNHGGGGNNSGPNSELNIYQYGGGNSALALQTDARNSDLTITQHGGGNGADVGQGSDDSSIDLTQRGFGNSATLDQWNGKNSEMTVKQFGGGNGAAVDQTASNSSVNVTQVGFGNNATAHQY

*_R5peptide_CsgA_CBD_*

SSKKSGSYSGSKGSKRRILGGGGSGGGGSGVVPQYGGGGNHGGGGNNSGPNSELNIYQYGGGNSALALQTDARNSDLTITQHGGGNGADVGQGSDDSSIDLTQRGFGNSATLDQWNGKNSEMTVKQFGGGNGAAVDQTASNSSVNVTQVGFGNNATAHQYGGGGSGGGGSAWQVNTAYTAGQLVTYNGKTYKCLQPHTSLAGWEPSNVPALWQLQHHHHHH

HydA.

MMHHHHHHENLYFQGMKTIILNGNEVHTDKDITILELARENNVDIPTLCF LKDCGNFGKC GVCMVEVEGKGFRAACVAKVEDGMVINTESDEVKERIKKRVSMLLDKHEFKCGQCSRRENCEFLKLVIKTKAKASKPFLPEDKDALVDNRSKAIVIDRSKCVLCGRCVAACKQHTSTCSIQFIKKDGQRAVGTVDDVCLDDSTCLLCGQCVIACPVAALKEKSHIEKVQEALNDPKKHVIVAMAPSVRTAMGELFKMGYGKDVTGKLYTALRMLGFDKVFDINFGADMTIMEEATELLGRVKNNGPFPMFTSCCPAWVRLAQNYHPELLDNLSSAKSPQQIFGTASKTYYPSISGIAPEDVYTVTIMPCNDKKYEADIPFMETNSLRDIDASLTTRELAKMIKDAKIKFADLEDGEVDPAMGTYSGAGAIFGATGGVMEAAIRSAKDFAENKELENVDYTEVRGFKGIKEAEVEIAGNKLNVAVINGASNFFEFMKSGKMNEKQYHFIEVMACPGGCINGGGQPHVNALDRENVDYRKLRASVLYNQDKNVLSKRKSHDNPAIIKMYDSYFGKPGEGLAHKLLHVKYTKDKNVSKHEGGGSGGGSAHIVM VDAYKPTK

HydE.

MDNIIKLINK AEVTHDLTKD ELVTLLKDDT HNEEIYKAADRVREKYVGEE VHLRGLIEFS NICKRNCMYCGLRRDNKNIKRYRLEPDEIIHLAKSAKNYGYQTVVLQSGEDDYYTVEKMKYIVSEIKKLNMAITLSIGEKTFEEYEEYRKSGADRYLIRIETTDKELYEKLDPKMSHENRINCLKNLRKLGYEVGSGCLVGLPNQTIESLADDILFFKEIDADMIGVGPFIPNEDTPLGEEKGGEFFMSVKVTALIRLLLPDINIPATTAMESLYPNGRSIALTSGANVVMPNVTEGEYRKLYALYPGKI CVNDTPGHCRQCISLKINKI NRKVSATKGFRKKSYKESIG

HydF

MNELNSTPKGERLHIALFGKTNVGKSSVINALTSQEIALVSNVKGTTTDPVYKAMELLPLGPVMLIDTAGLDDISDLGELRRGKTLEVLSKTDVAILVFDVESGITEYDKNIYSLLLEKKIPLIGVLNKIDKKDYKLEDYTSQFKIPIVPISALNNKGINNLKDELIRLAPENDDKFKIVGDLLSPGDIAVLVTPIDKAAPKGRLILPQQQTIRDILESDAIAMVTKEFELRETLDSLRKKPKIVITDSQVFLKVAADTPKDILMTSFSILMARHKGDLIELARGARAIEDLKDGDKILIAEACTHHRQSDDIGKVKIPRWLRQKTGKKLEFDFSSGFSFPPNIEDYALIVHCAGCMLNRRSMLHRIESSVKKQIPIVNYGVLIAYVQGI LPRALKPFPYADRIFNQSSRN

HydG

MYNVKSKVATEFISDEEIIDSLEYAKQNKSNRELIDSIIEKAKECKGLTHRDAAVLLECDLEDENEKMFKLAREIKQKFYGNRIVMFAPLYLSNYCVNGCVYCPYHHKNKHIARKKLSQEDVKRETIALQDMGHKRLALEAGEDPVNNPIEYILDCIKTIYSIKHKNGAIRRVNVNIAATTVENYKKLKDAGIGTYILFQETYNKKSYEELHPTGPKHDYAYHTEAMDRAMEGGIDDVGIGVLFGLNMYKYDFVGLLMHAEHLEAAMGVGPHTISVPRIRPADDIDPENFSNAISDEIFEKIVAIIRIAVPYTGMIVSTRESKKTRERVLELGISQISGGSSTSVGGYVESEPEEDNSSQFEVNDNRTLDEIVNWLLEMNYIPSFCTACYREGRTGDRFMSLVKSGQIANCCQPNALMTLKEYLEDYASSNTQKNGEALIASEVEKIPNEKVKSIVKKHLTELKEGQRDFRF

**3 Gene construction**

Recombinant constructs for the triple-domain fusion proteins (CsgA protein fused with a C-terminal CBDs and an N-terminal R5 peptide, appended with a C-terminal poly-histidine tag) were generated using isothermal Gibson assembly, followed by cloning into the pET-22b expression vector. The sequences of the constructed plasmids were confirmed by restriction enzyme digestion, and sequencing was performed commercially by Genewiz (Suzou). Construction of hydrogenase gene cluster: a single-fusion plasmid containing co-expressed genes of the hydrogenase A (HydA) and its maturases (HydE, HydF and HydG) was created by leveraging the ACEMBL expression system for multigene expression [1, 2]. This multigene plasmid was expressed in BL21(DE3) cells to generate the hydrogenase-producing bacteria referred to as E. coli BL21(DE3)/pAEFG[3].

**4 Detailed protocols for SDS/PAGE and Western blotting SDS/PAGE**

SDS/PAGE: Samples were mixed with loading sample buffer (Life Technology) in 1.5 mL microtubes. Afterwards, the mixed solution together, along with the standard protein ladder (Life Technology), were then loaded into the lanes of the mini gels (Life Technology), which were run at 165 volts for 35 mins using Nupage MES SDS running buffer (NOVEX) Following this step. The gels were then stained with Coomassie Blue for1 h and then destained with washing solution (10% acetic acid / 40% Methanol / 50% DI water) for 1 h twice. The gels were imaged using a Bio-Rad ChemiDoc MP system.

Western blotting: Samples were separated in12% SDS-polyacrylamide gels and blotted onto polyvinylidene difluoride membranes using iBlot system (Invitrogen). Membranes were incubated with 30 mL monoclonal mouse antibodies (LifeTein) at a dilution of 1:5000 for 1 h and washed with 10 mL 1 × TBST for 10 min for three times. They were then incubated with 30 mL secondary goat antimouse antibodies IgG conjugated to horseradish peroxidase (Sigma) at a dilution of 1:5000 for 1 h. After treatment with 1 × TBST, the membranes were imaged through a Bio-Rad ChemiDoc MP system

**5. Preparation of functional CsgA fibers and functional CsgA fibers/chitin ink**

*Functional CsgA fiber ink*

Freshly purified control and functionalized CsgA fibers were incubated at room temperature overnight. The solutions containing self-assembled nanofibers were subjected to dialysis (3 kDa, Yeasen) with double distilled water (ddH_2_O) for 72 h to remove salts, followed by lyophilization. The dry powders were then dissolved in HFIP (2 mg/mL) with occasional shaking to allow complete dissolution. Before use, the solutions were filtered with a 220 nm PTFE filter (Millipore); the typical dissolution process lasted for one week. HFIP is a toxic and volatile solvent, so all of the procedures were performed in a fume hood.

*Functional CsgA fibers /chitin hybrid ink*

Functional CsgA fibers/chitin hybrid ink was prepared by mixing functional CsgA fiber ink and chitin ink at a weight ratio of 1:1 (total 2 mg/mL). Briefly, β-chitin powder (Industrial Research Ltd of New Zealand) was completely dissolved in HFIP (2 mg/mL). Before use, the functional csgA fibers/chitin hybrid ink solution was filtered with a 220 nm PTFE filter (Millipore).

**6. Morphology characterization**

TEM: Bright-field TEM images were obtained on an FEI T12 transmission electron microscope operated at 120 kV accelerating voltage, high-angle annular dark-field imaging (HAADF) were performed on a JEM-F200 electron microscope operating at 200 kV accelerating voltage after staining the samples with 7 µL 2 wt% uranyl acetate for 1 min. AFM: AFM images were collected on an Asylum MFP-3D or Bruker Fast Scan AFM using the tapping mode with AC160TS-R3 cantilevers (Olympus, k ≈ 26 N/m, ν≈300 kHz). SEM: For SEM, the samples were washed with deionized water, dried under a constant flow of N2, and coated with Au (sputtered to ~10 nm) with a SBC-12 sputter coater. The samples were then imaged a JSM 7800 scanning electron microscope operated at 5 kV accelerating voltage.

**7. Chemical composition characterization**

EDS: EDS were performed on a JEM-F200 electron microscope operating at 200 kV accelerating voltage. Nano-IR: The IR chemical mappings were acquired using a nano-IR system (Anasys Instruments), which acquired at wavenumber of 750 cm^−1^ assigned to the Ti-O stretch absorption. The gold-coated AFM tips had about 250 kHz resonance (Tap300G-B-G, budgetsensors.com). XPS: XPS was conducted by using an ESCALAB 250Xi (Thermo) with a monochromatic Al-Kα line (1486.6 eV). Elements of samples’ surfaces were measured at a step width of 0.01 eV.

**8. Porous patterned sheet fabrication via a microtransfer molding process**

PDMS stamps were fabricated by casting mixed pre-polymer and curing agent using commercial porous patterned silicon structure (Yuanwei Nano, Suzhou) as the initial mold; SYLGARD 184 was used with a 10:1 polymer base curing agent ratio. After pouring over the master and vacuum degassing, the PDMS was cured at 70°C for 4 h and lifted out. After curing, PDMS was cut into the desired defined shape with a razor blade. Using a common drop-casting process, 200 μL PVA (5%) aqueous solution was then cast onto a glass substrate (1cm × 1cm) to form a uniform sacrificial layer. Self-supporting PPS were prepared after dissolving the sacrificial layer with copious amount of ddH_2_O following the same microtransfer molding and curing processes. After peeling off the PDMS stamps, the samples were placed in a sealed chamber with a methanol atmosphere, created by bubbling N_2_ gas through a flask containing methanol solvent. The curing process in methanol gas was carried out for at least one week. The samples were then immersed in methanol overnight for final curing. The obtained PPS was washed with deionized water for three times, and then either freeze-dried with liquid nitrogen or left to dry overnight in the air. The sample was then used for further experiments in this study.

**9. Biomimetic mineralizaton**

Silicification Reactions: for the nanofiber samples, 200 µL of 1 M tetramethoxysilane hydrolyzed in 1 mM HCl was then added to 2 mg/mL fibers dissolved in phosphate buffer and shaken for 10min. The samples were centrifuged for 5 min at 10,000 rpm and washed three times with deionized water. And for the porous patterned sheets (PPS), a total of 200 µL of 100 mM phosphate buffer was added to cover the air-dried porous protein films, and 20 µL of 1 M tetramethoxysilane hydrolyzed in 1 mM HCl was then added to initiate the silicification reaction; the reaction was left to incubate for 10 min. The sheets were then washed three times with water (resistance 18.2 MΩ) and left to dry overnight in the fume hood. Mineralization of Titanium Dioxide: for the nanofiber samples, Titanium(IV) bis(ammonium lactato) dihydroxide (20 µL, 1 M) was added to 200 µL of 2 mg/mL fibers dissolved in phosphate buffer and shaken for 1 h. The samples were centrifuged for 5 min at 10,000 rpm and washed three times with deionized water. For air-dried PPS, the samples were covered in 1.5mL polyethylene micro-centrifuge tubes with 1 mL 1 M Titanium(IV) bis(ammonium lactato) dihydroxide for 1h at room temperature. The sheets were then washed three times with water (resistance 18.2 MΩ) and left to dry overnight in the fume hood. Mineralization of Gallium Oxide: air-dried PPS or 2mg/mL fibers were incubated in 1.5mL polyethylene micro-centrifuge tubes with 1 mL of a 1.0 M aqueous solution of gallium nitrate (GNO, Alfa) for 6h at 25°C. The samples were centrifuged for 5 min at 10 000 rpm and washed three times with alcohol and deionized water and left to dry overnight in the fume hood. All other experiments were conducted at a pH of 7.5.

**10. The sugar fab process for the mineralized cubes**

The cube assisted by cubic-like porous sugar (Taikoo) templates was made using 3D soft lithography. About 0.6~0.8 mL 2 mg/mL CsgA /chitin /HFIP solution was cast into the 3D sugar template. A mild vacuum was applied during casting to fully ﬁll the interspaces of the bonded sugar template with solution. After the HFIP was evaporated, the samples were cured under methanol vapor for 4 days at 20 °C, and then the sugar template was leached away using distilled water, resulting in a porous cube structure. To obtain mineralized TiO_2_ cubes, a similar biomimetic mineralization process under the aforementioned mineralization conditions was performed in the presence of the fabricated porous cube structures

**11. Photocurrent detection from the mineralized cubes**

Both mineralized and unmineralized cubes were spread onto an 1 × 1 cm FTO conductive glass and dried in an incubator at 37 °C overnight, and then a three-electrode system was used for photocurrent testing. A platinum wire and an Ag/AgCl were used as counter and reference electrodes, respectively. The applied potential was 0 V, and the reaction solution was 0.5 M Na_2_SO_4_. The illumination intensity was 20.07 mW/cm^2^. The solution was purged with continuous N_2_ bubbles for 30 min to get rid of oxygen in the electrolyte cell.

**12. Cyclic voltammetry measurement**

Cyclic voltammetry test of TiO_2_-mineralized cube was carried out at -0.1 V to 1.4 V versus RHE using a three-electrode configuration in fresh 0.50 M aqueous Na_2_SO_4_ electrolyte at pH 6.3.

**13. Hydrogen-evolution experiment**

The fabricated dehydrated TiO_2_ mineralized porous cubes were first immersed into PBS solution containing excessive E. *coli* BL21 (DE3)/pAEFG cells collected from 10 mL culture to allow sufficient absorption of the engineered strains into the cube. The cube was then moved into a 4-mL bottle containing the hydrogen evolution reaction system composed of 2.6 mL mixed solution of TEOA (1.5 %), MV (5 mM), and glycerol (5 %) in PBS solution (pH = 8.0). Triethanolamine (TEOA) and methyl violet (MV) were supplemented into the system as a sacrificial electron donor and mediator, respectively. Afterwards the hydrogen production was proceeded upon exposure of the reaction system to a 300 W Xenon lamp (CEL-HXF300, CEAULIGHT) with the illumination intensity of 20.07 mW/cm^2^. Afterwards the hydrogen production was induced upon exposure of the reaction system to a collimated 300 W Xenon lamp (CEL-HXF300, CEAULIGHT.), the light emitted is a continuous spectrum with an energy distribution from 350 nm to 1200 nm, which is similar with the sunlight energy distribution. Headspace gas was periodically monitored by an Agilent 3000A Micro gas chromatography (Ar carrier gas, molecular sieve column, thermal conductivity detector).

**14. X-ray fiber diffraction of amyloid nanofibers**

To investigate the β structure, nanofibers formed by proteins were pelleted by centrifugation at ~12,000 × g, followed by washing several times with copious amounts of ddH_2_O to remove salts. The nanofiber pellets were then suspended in ddH_2_O. Two microliter suspensions were pipetted between two fire-polished glass rods and dried for several hours. The diffraction data was collected on a Rigaku Micromax- 007 X-ray generator and an R-Axis IV++ area detector.

**15. Quantitative assessment of Young’s modulus by PK-QNM**

Samples were rinsed and dried, followed by AFM (Bruker Fast Scan) in peak force quantitative nanomechanics (QNM) mode with an RTESPA-100 probe (40N/m, 300kHz, Rotated Tip, Al Reflective Coating). Values of Young’s modulus can be extracted from the DMT modulus channel.[4, 5]In the DMT model:

$$F=\frac{4}{3}\frac{E}{(1-\mu^{2})}\sqrt{R}\delta^{\frac{3}{2}}$$

Where F is the force that can be acquired from the fitting force curve; E is the Young’s modulus; μ is the Poisson’s ratio; R is the radius of tip; δ is the indentation depth. Sample preparation for Young’s modulus measurements:

For the patterned sample which were directly applied on a silicon substrate (~ 1cm^2^ ) and dried in a sealed container. The deflection sensitivity of cantilevers (AC160TS-R3, Olympus, k ≈ 26 N/m, ν ≈ 300 kHz) was calibrated on a mica substrate. Tip radius was measured using a tip characterizer sample (Bruker). Deformation was kept in the range of 5–10 nm. For a specific area of the sample, 256 × 256 points were tested to obtain corresponding force curves. The values of F and δ could be acquired from these force curves. The Poisson’s ratio of the uncured and cured proteins was set for 0.3. The calculations of Young’s modulus were performed using Nanoscope Analysis software. All the values of Young’s modulus were exported and plotted using Origin software.

**16. Nano-indentation for the Young’s modulus assessment**

Samples were placed on glass coverslips and tested in ddH_2_O, using a displacement-controlled PIUMA Nanoindenter (Optics11 B.V., Amsterdam, The Netherlands). A probe having a 0.47 N/m cantilever stiffness (k) and a 52.0 µm radius (R) spherical tip was used to measure the mechanical properties. Nano-indentation measurements were started out of sample contact and performed on different surface points (randomly selected) to avoid sample pre-stress and any eventual effect due to repeated testing cycles on the same spot, respectively. The most commonly used method, developed by Oliver and Pharr, estimates the contact area from the contact depth, determined from the load-displacement curves[6]. The measurement was recorded for 5000 nm maximum depth (thus a typical rough loading curve was observed for the microporous scaffolds).

**Supplementary Figures:**


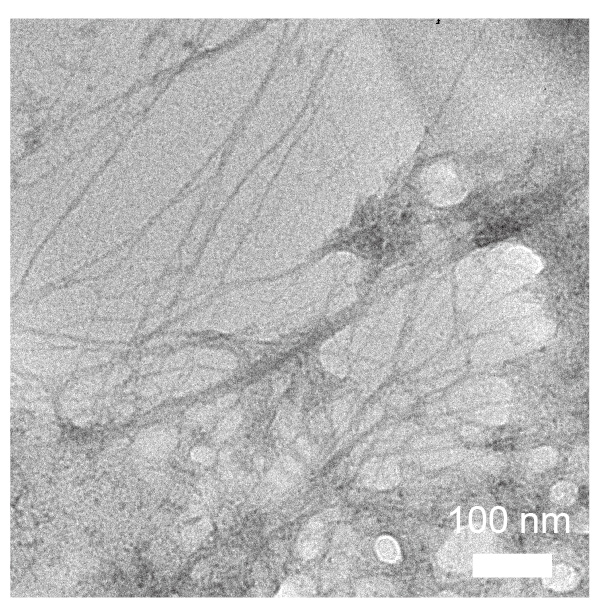


**Supplementary Figure 1. Morphological characterization of the _R5_CsgA nanofibers.** TEM image of _R5_CsgA nanofibers.


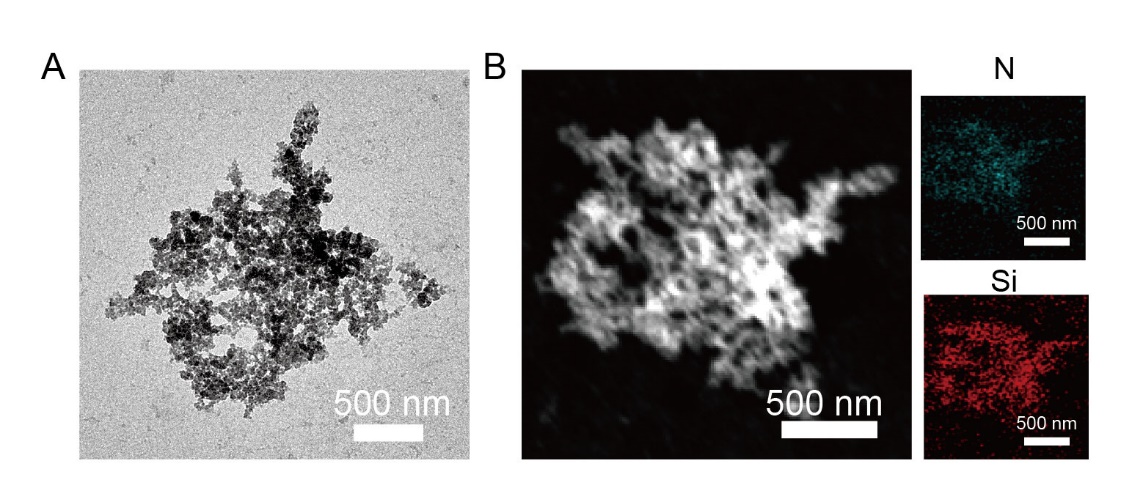


**Supplementary Figure 2. Morphological and chemical composition characterization of _R5_CsgA nanofibers after SiO_2_ mineralization.** (A) TEM image of the _R5_CsgA nanofibers after mineralization of SiO_2_. (B) STEM-EDS images of the _R5_CsgA nanofibers after mineralization of SiO_2._

_._


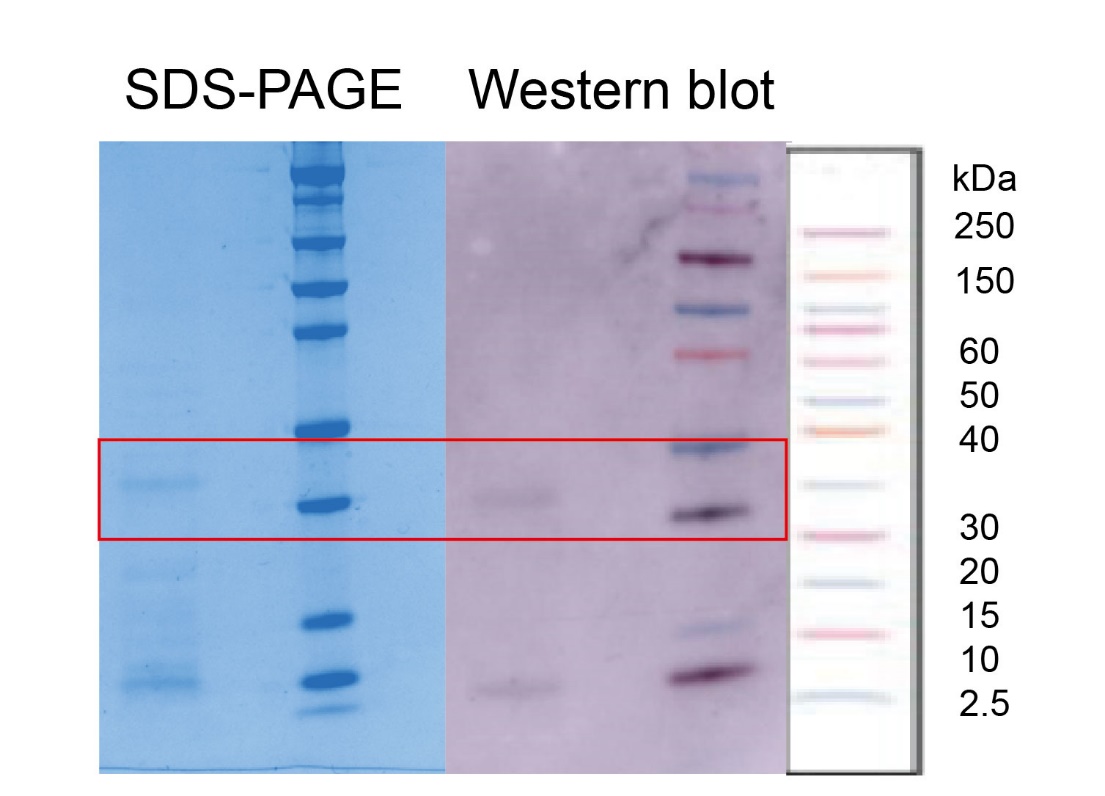


**Supplementary Figure 3.** Coomassie-stained SDS-PAGE gels and western blotting using anti-His antibodies to detect the recombinant cobalt-resin column-purified _R5_CsgA_CBD_ proteins.


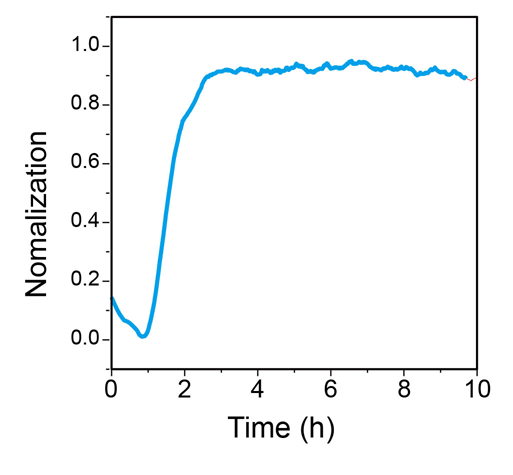


**Supplementary Figure 4.** ThT assay showing the kinetics of amyloid formation of _R5_CsgA_CBD_ proteins.


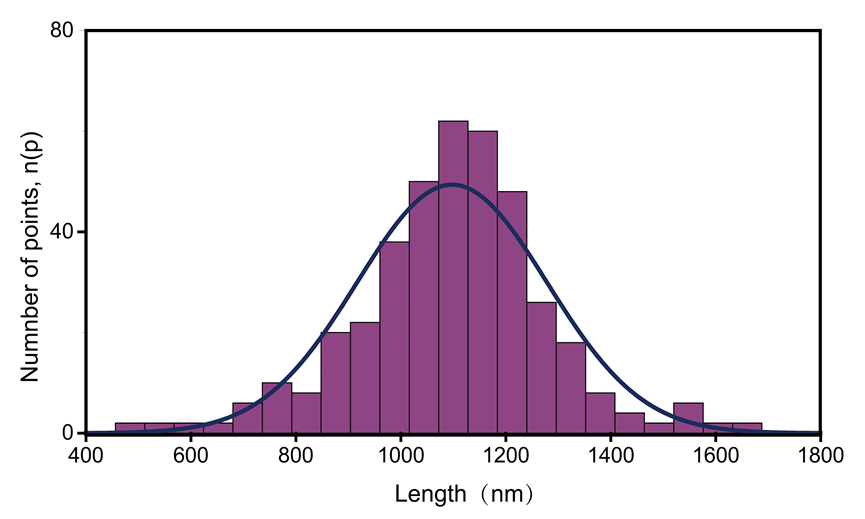


**Supplementary Figure 5.** Statistical analysis of the lengths of _R5_CsgA_CBD_ fibrils based upon AFM height images. The number of measured points (Y axis) as a function of given length range (X axis) were plotted. After fitting with Gaussian function, length of fibrils was calculated. N= 400 random fibrils.

_
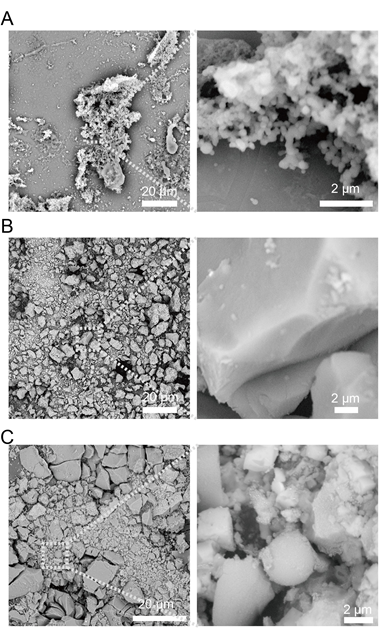
_

**Supplementary Figure 6. SEM images of mineralized silica structures formed in the presence of different protein nanofibers:** (A) _R5_CsgA_CBD_; (B) CsgA; (C) CsgA_。_ These results highlight the obvious mineralization-promoting effect of R5 peptide in the designed fusion protein.


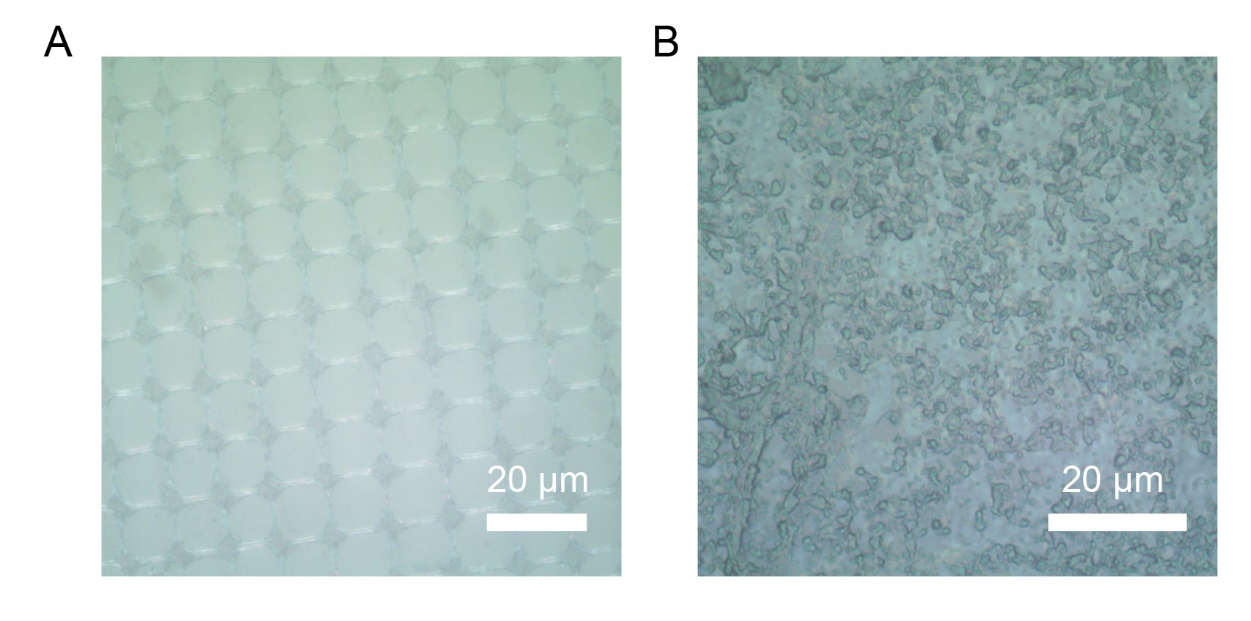


**Supplementary Figure 7. Optical microscopy images of self-supporting patterned porous sheets composed of different components.** (A) _R5_CsgA_CBD_ and chitin nanofibers and (B) _R5_CsgA_CBD_ nanofibers after thorough immersion in aqueous solution. Note that the sheet made from _R5_CsgA_CBD_ exhibited insufficient cohesion to maintain itself as a self-supporting pattern.


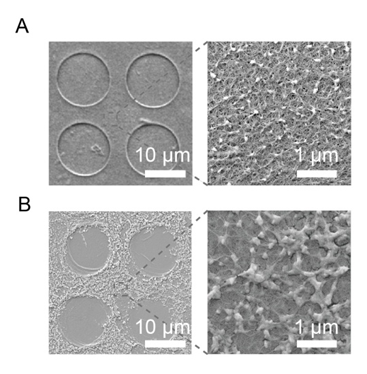


**Supplementary Figure 8. morphological characterization of silicificated porous sheets composed of** **different compounds.** SEM images of mineralized chitin (A) and mineralized complex PPS structures (B) after 10-min mineralization.

**
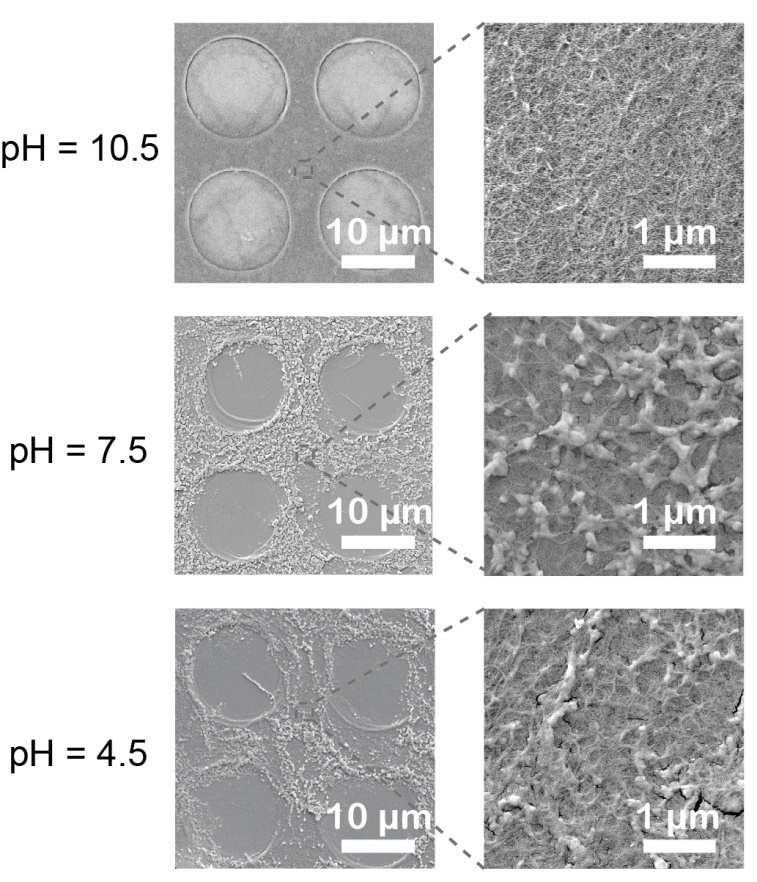
Supplementary Figure 9.** SEM images of mineralized silica structures formed in solutions with different pH values.


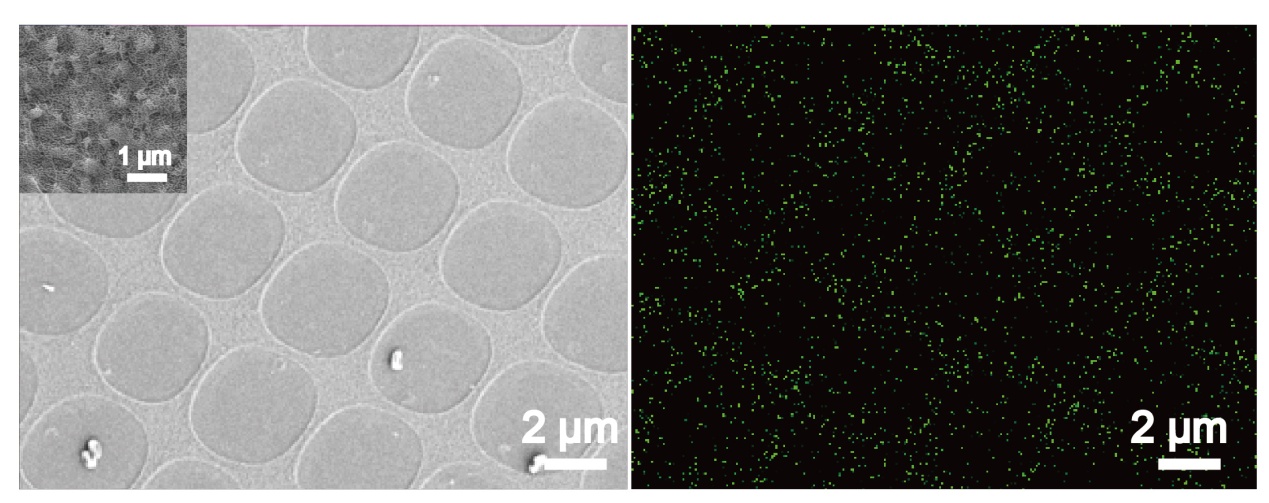


**Supplementary Figure 10.** SEM image and the corresponding EDS image of the TiO_2_-mineralized PPS to reveal the presence of Ti element on the surface. The insert in the left image represents a zoomed-in area on the PPS.

**
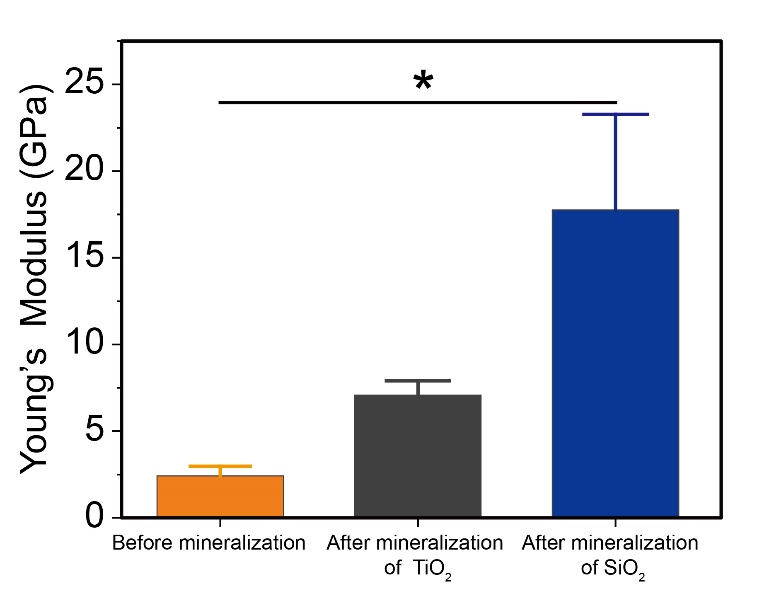
**

**Supplementary Figure 11.** The Young’s modulus of R5CsgACBD/chitin PPS before and after mineralization of TiO_2_ and SiO_2_ using peak force quantitative nanomechanical AFM methodology. *P < 0.05, student's *t*-test. Note：The data was obtained by statistics from 256 × 256 spots in a 4 μm^2^ square per sample.


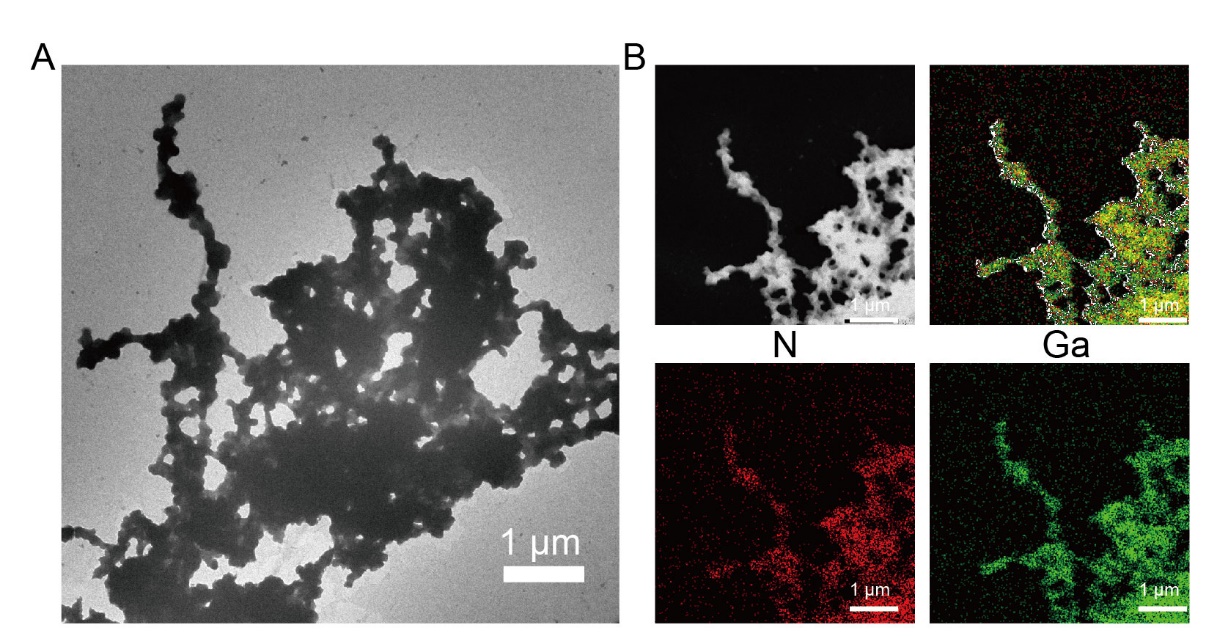


**Supplementary Figure 12. Morphological and chemical composition characterization of _R5_CsgA_CBD_ nanofibers after mineralization of Ga_2_O_3._** (A)TEM image of the _R5_CsgA_CBD_ nanofibers after mineralization of Ga_2_O_3_. (B) STEM-EDS images of the _R5_CsgA_CBD_ nanofibers after mineralization of Ga_2_O_3._


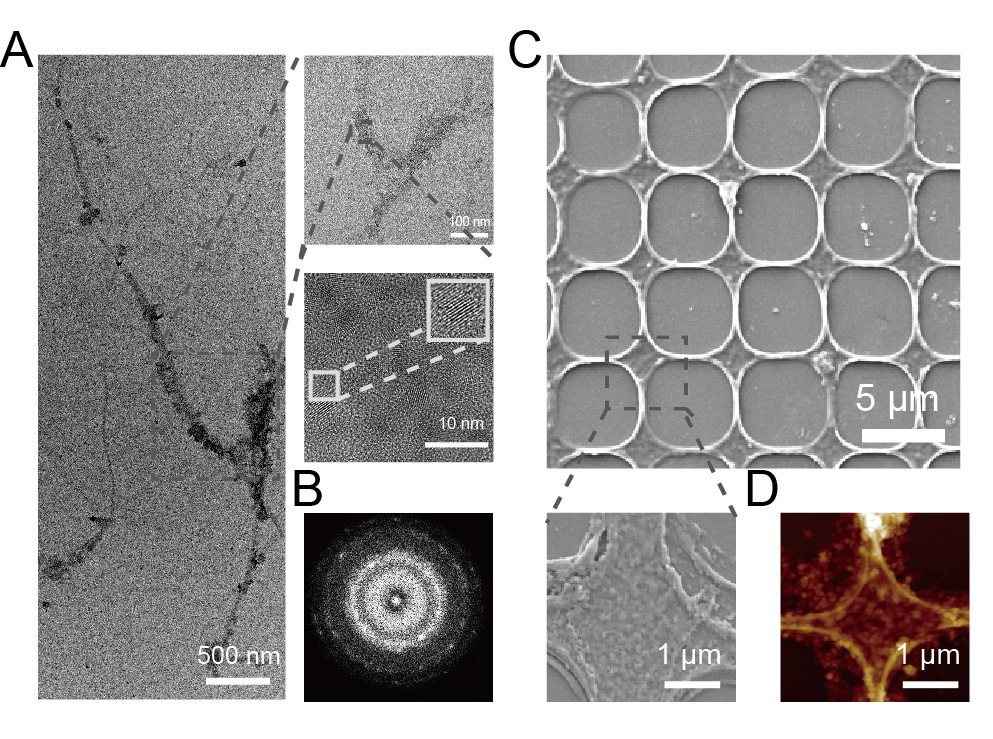


**Supplementary Figure 13. Morphological and structural characterization of _R5_CsgA_CBD_ nanofibers after mineralization of Ga_2_O_3_.** (A) TEM images and expanded view of lattice fringes by HR-TEM illustrating (311) planes of Ga_2_O_3_ crystals. (B) Diffraction patterns corresponding to Ga_2_O_3_ mineral are assigned. (C) SEM images of the Ga_2_O_3_ mineralized PPS. (D) AFM height image of the mineralized patterned porous sheets after Ga_2_O_3_ mineralization.


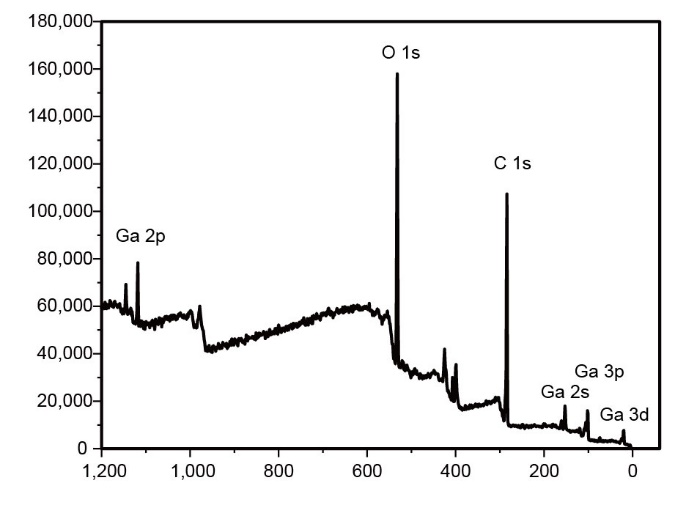


**Supplementary Figure 14.** The chemical identity of the Ga_2_O_3_ coating on the _R5_CsgA_CBD_ PPS were verified by XPS survey spectra.

**
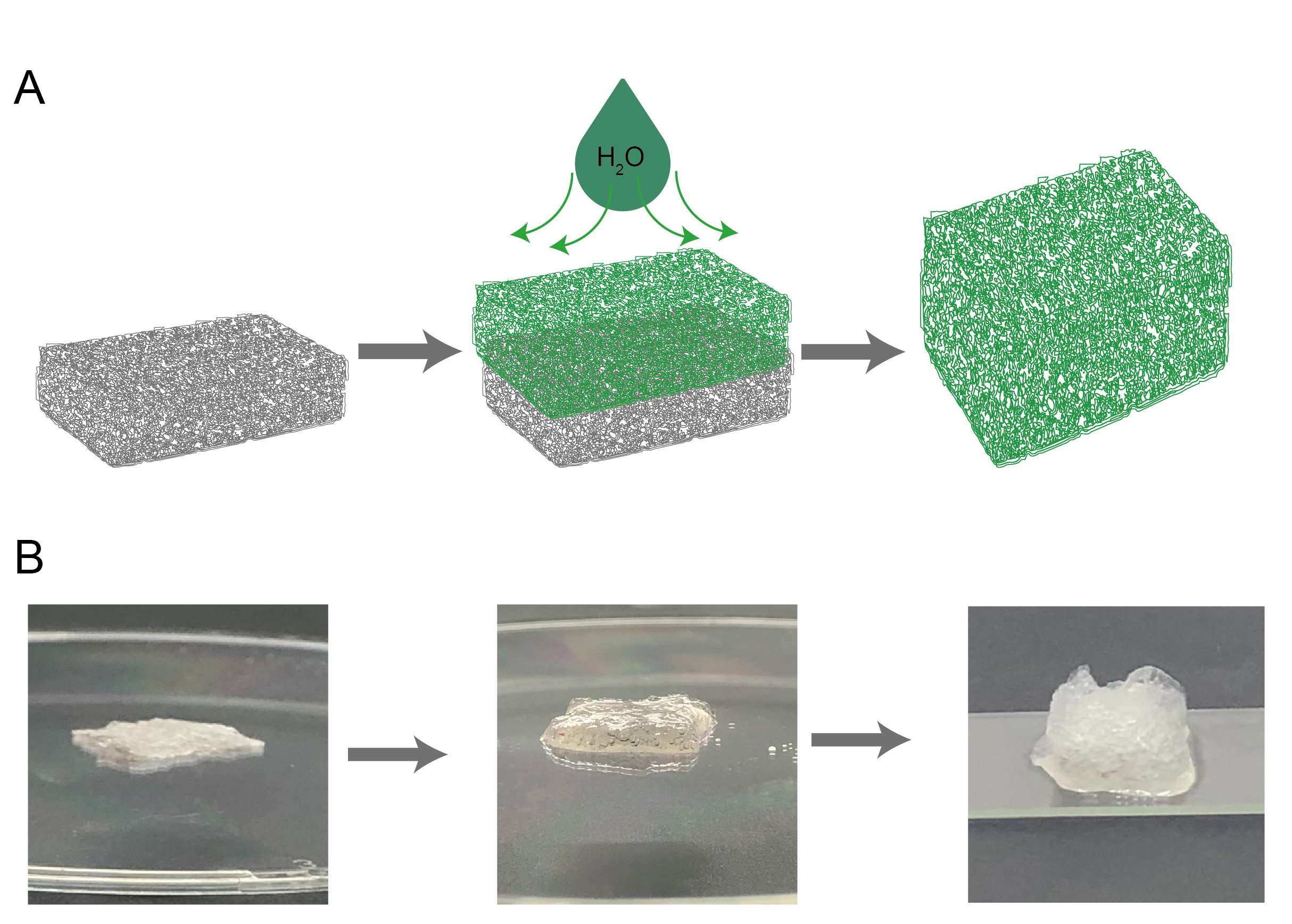
**

**Supplementary Figure 15. Morphology changes of the porous _R5_CsgA_CBD_/chitin cube from dehydrated to hydrated state.** (A) Schematic illustration of the morphological changes between the two states. (B) Corresponding photographic images.

**
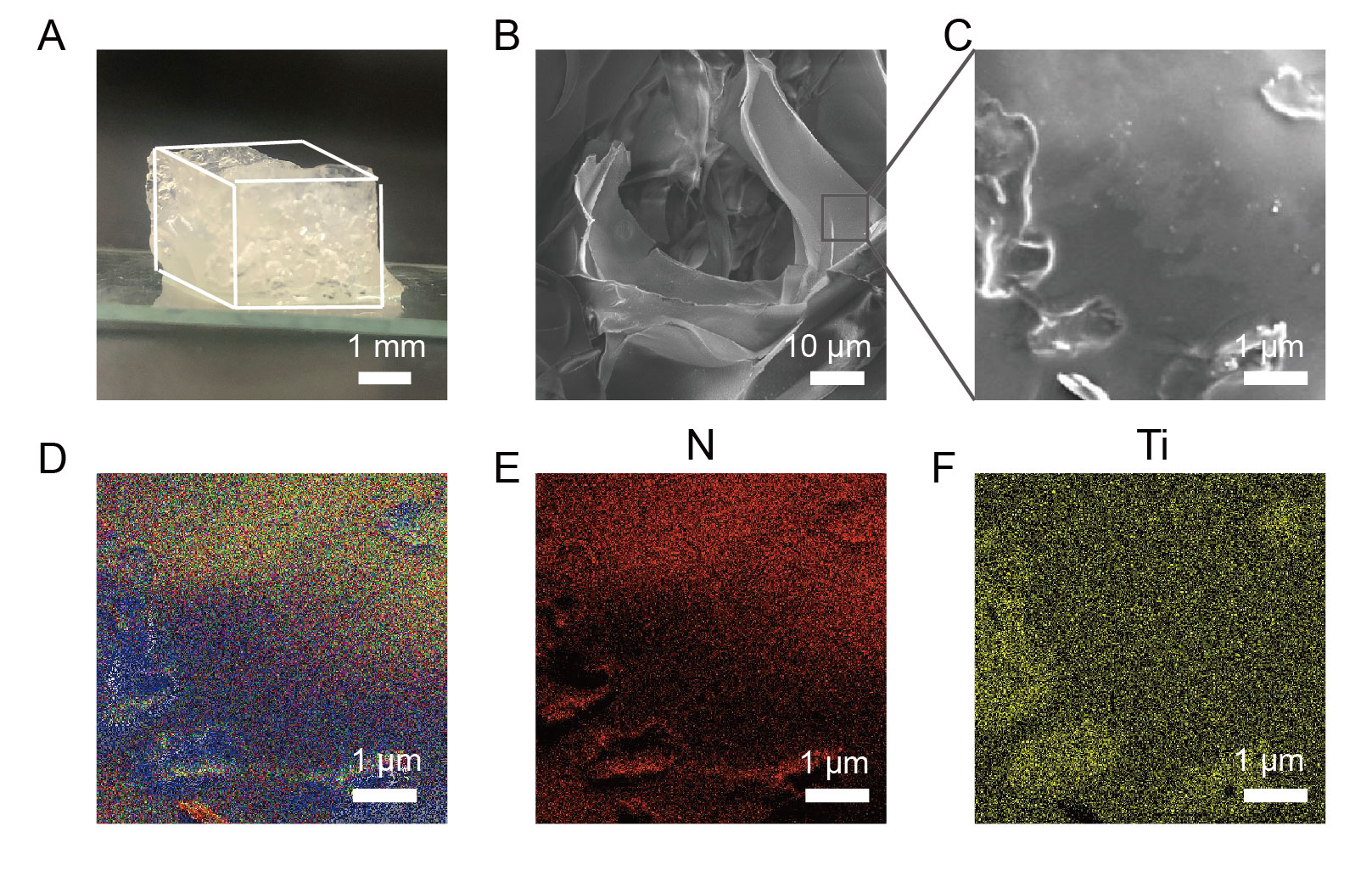
**

**Supplementary Figure 16. Morphological characterization and chemical compositions of the mineralized porous _R5_CsgA_CBD_/chitin cube.** (A) Photograph images of mineralized porous _R5_CsgA_CBD_/chitin cubes. (B) SEM image of mineralized porous _R5_CsgA_CBD_/chitin cubes. (C) SEM image of a zoomed-in area from (B). (D-F) SEM-EDS images of the _R5_CsgA_CBD_ nanofibers after mineralization of TiO_2._ The EDS analysis indicated the presence of Ti on the surface of the cube after mineralization (E) and the presence of N element from the proteinaceous components (F). Note: The blue color represents the element exists of oxygen.

**
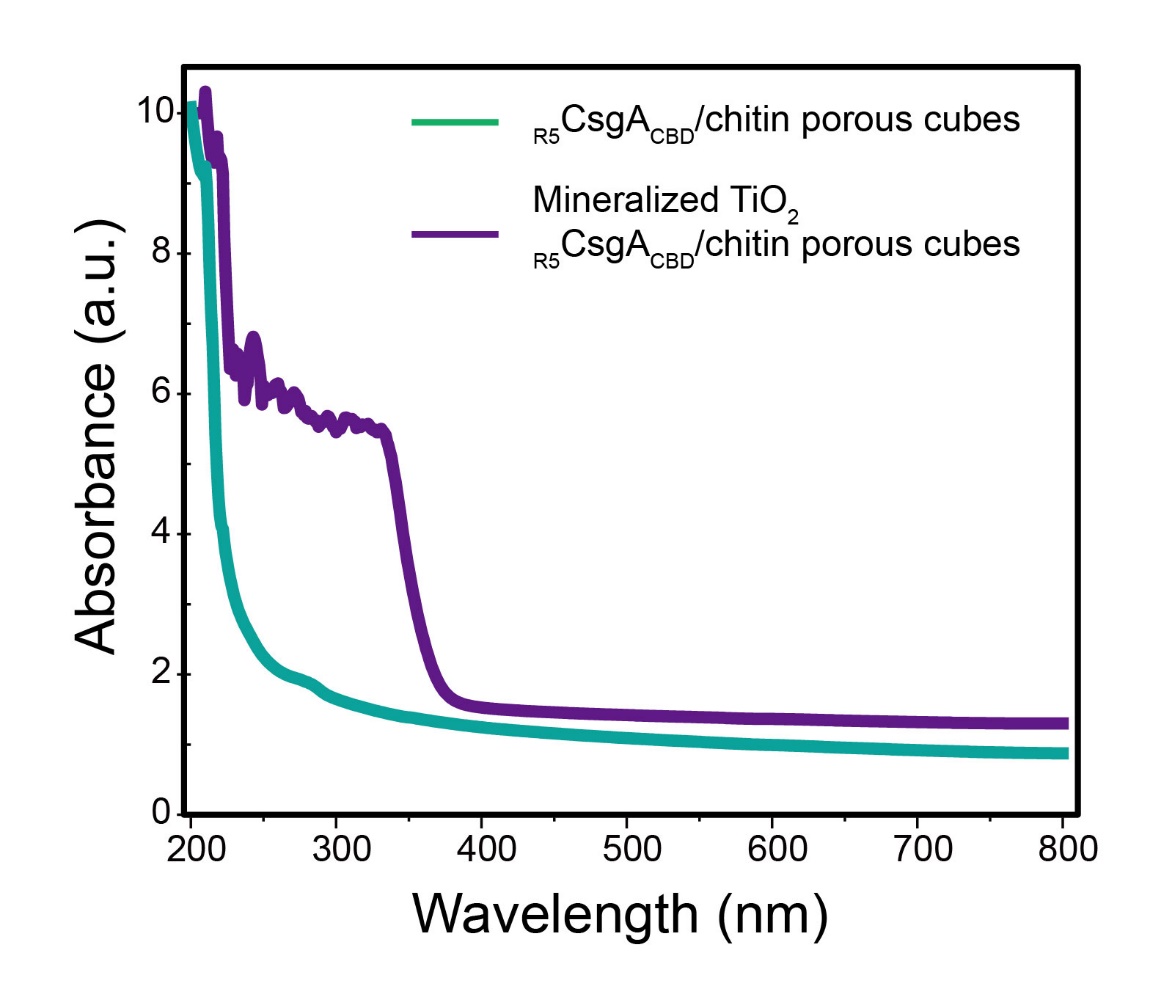
**

**Supplementary Figure 17.** UV−vis spectra of _R5_CsgA_CBD_/chitin porous cubes and TiO_2_-mineralized _R5_CsgA_CBD_/chitin porous cubes.

**
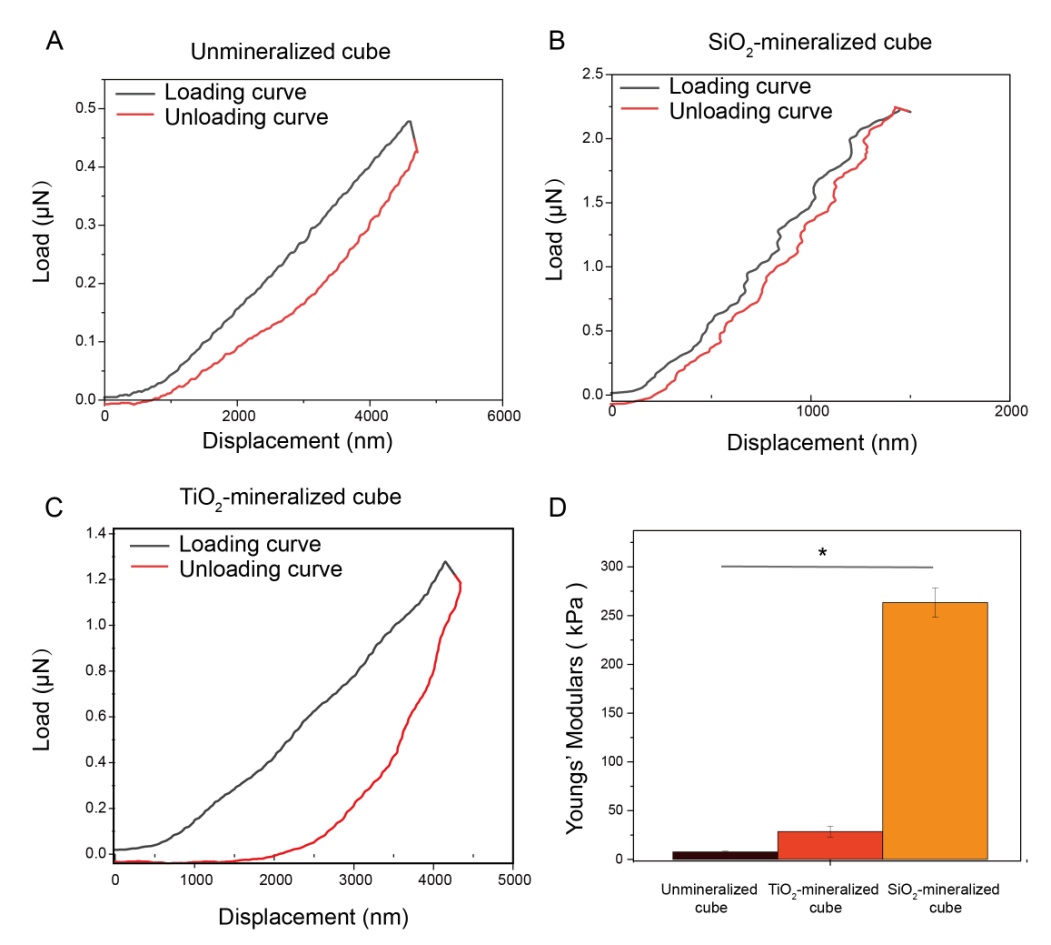
Supplementary Figure 18. Young’s modulus of _R5_CsgA_CBD_/chitin porous cube before and after mineralization of TiO_2_ and SiO_2_.** (A-C) Nanoindentation load-displacement curves for unmineralized, SiO_2_-mineralized and TiO_2_-mineralized cube. (D) Comparison of young’s modulus of this three samples. *P <0.05 student’s *t*-test N = 11.

**
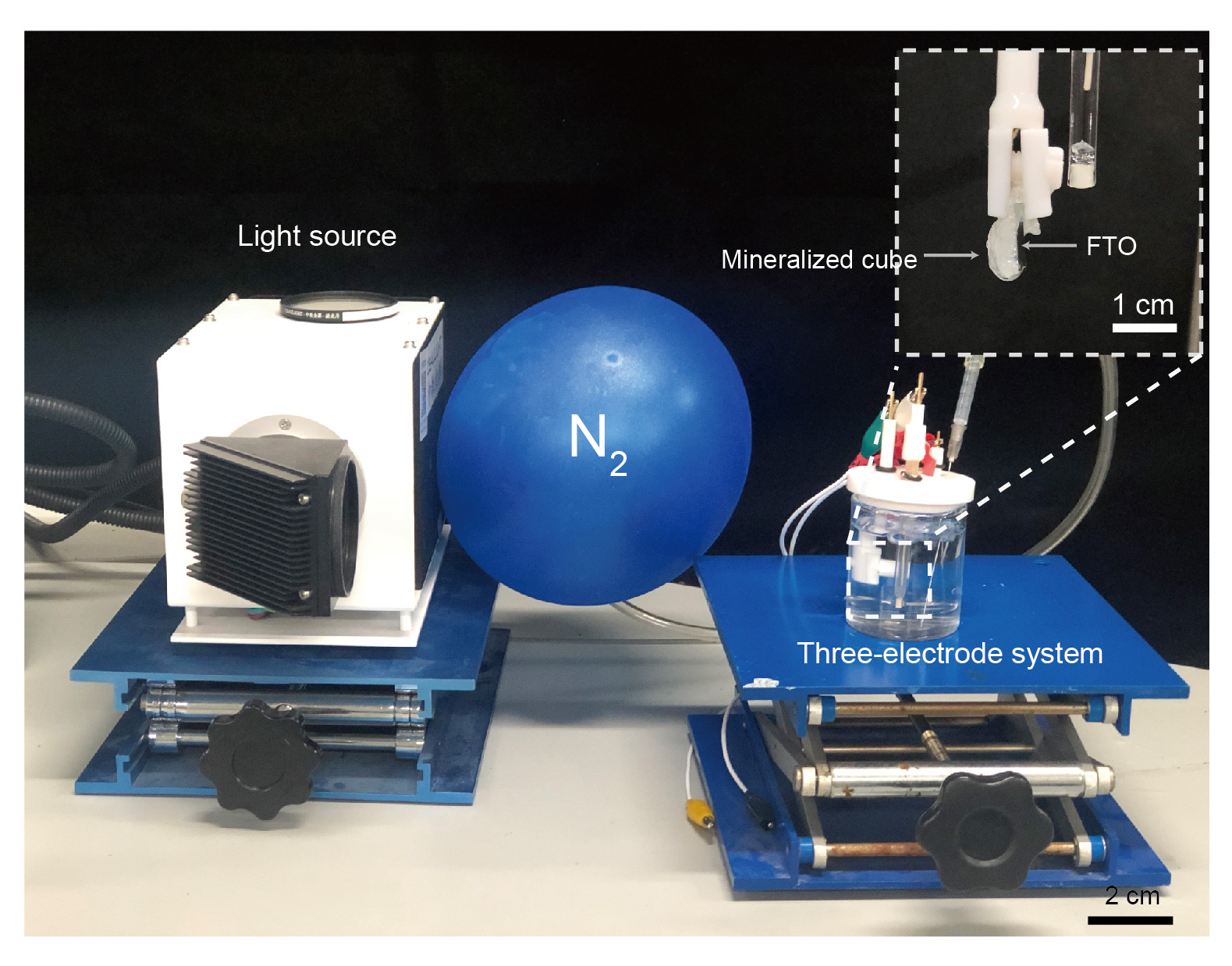
**

**Supplementary Figure 19.** The photographs of the (photo)electrochemical experiments equipment which were carried out with a typical three-electrode system using the prepared FTO with mineralized amyloid cube as working electrode, a platinum counter electrode and an Ag/AgCl (in aqueous KCl, 3.0 M) reference electrode.

**
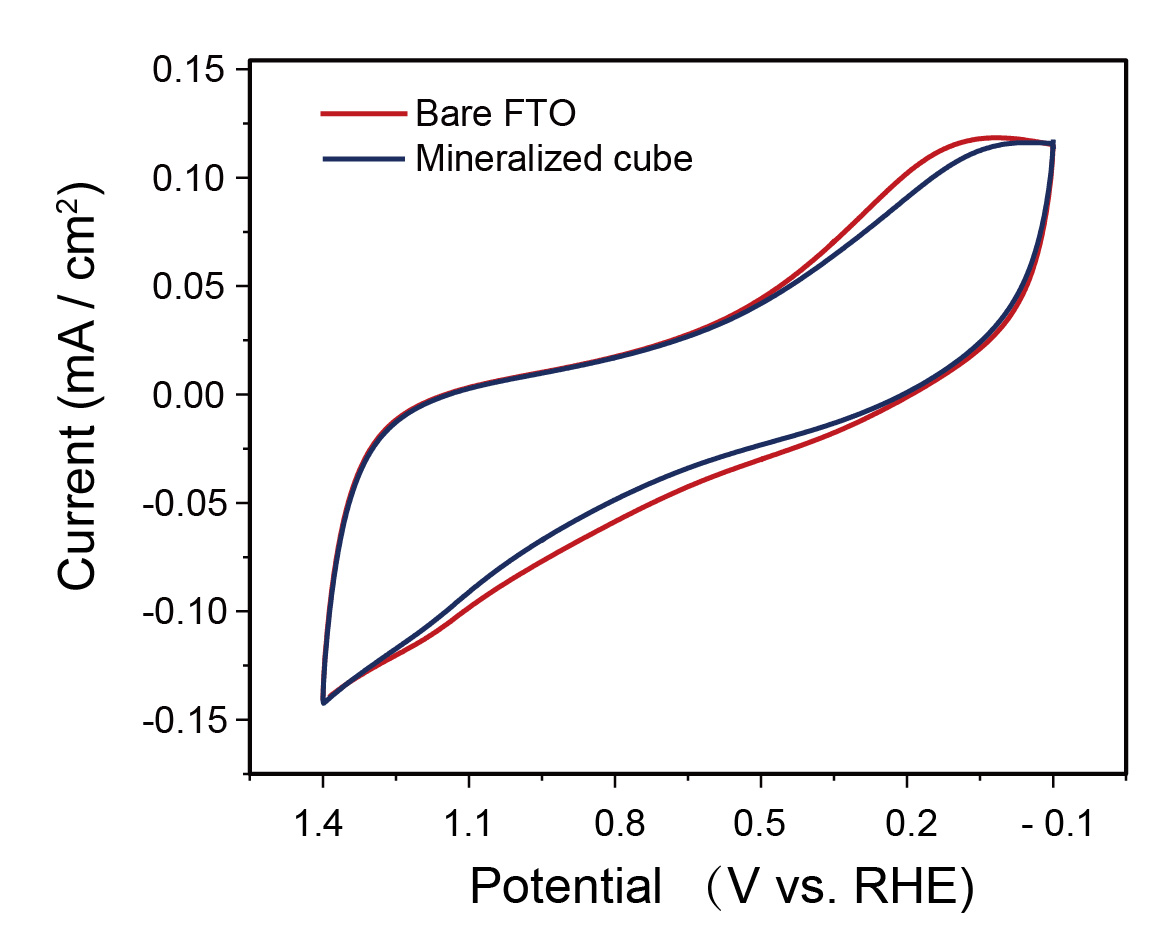
**

**Supplementary Figure 20.** Cyclic voltammograms of a bare FTO substrate and of the prepared FTO with mineralized amyloid cube (in aqueous 0.5 M Na_2_SO_4_ electrolyte (pH = 6.3)). Note: The experiments were carried out at -0.1 V to 1.4 V versus RHE using a three-electrode configuration.

**
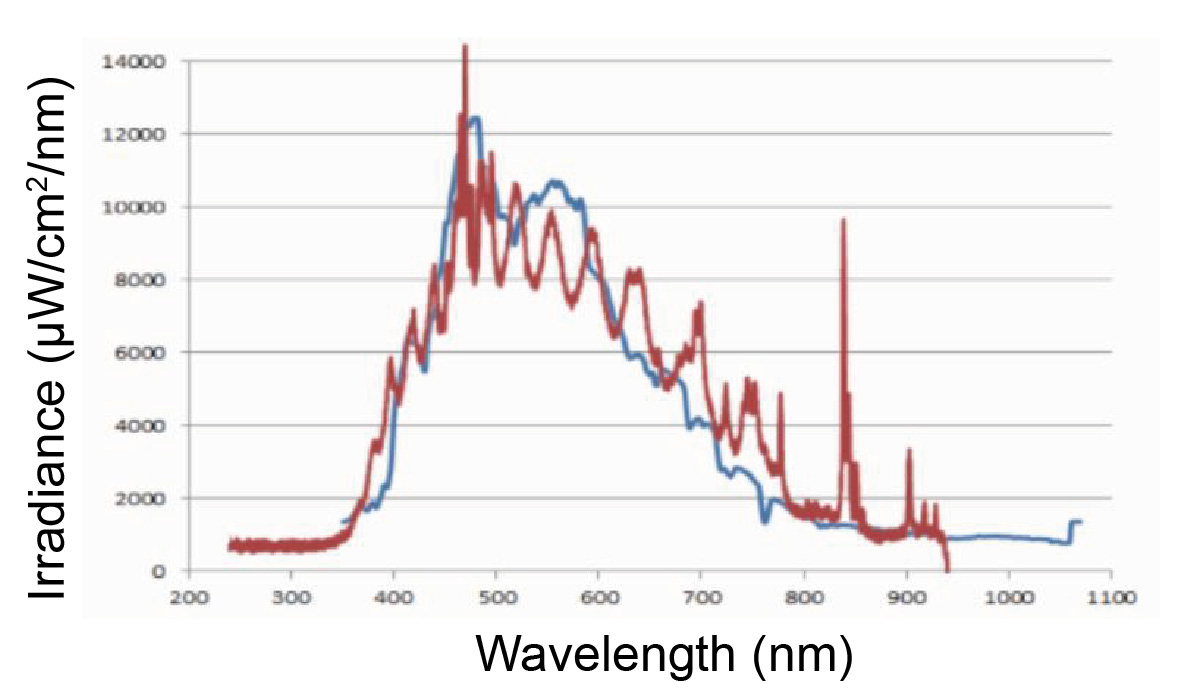
**

**Supplementary Figure 21.** Optical spectra of Xe lamp (red curve) and sunlight (blue curve). Note: the photograph image of hydrogen evolution equipment for the artificial photosynthesis systems based on mineralized porous cube structures was showed in the main text Figure 5B. These spectra were copied from the Instructions of the Xenon lamp (CEL-HXF300, CEAULIGHT).

**
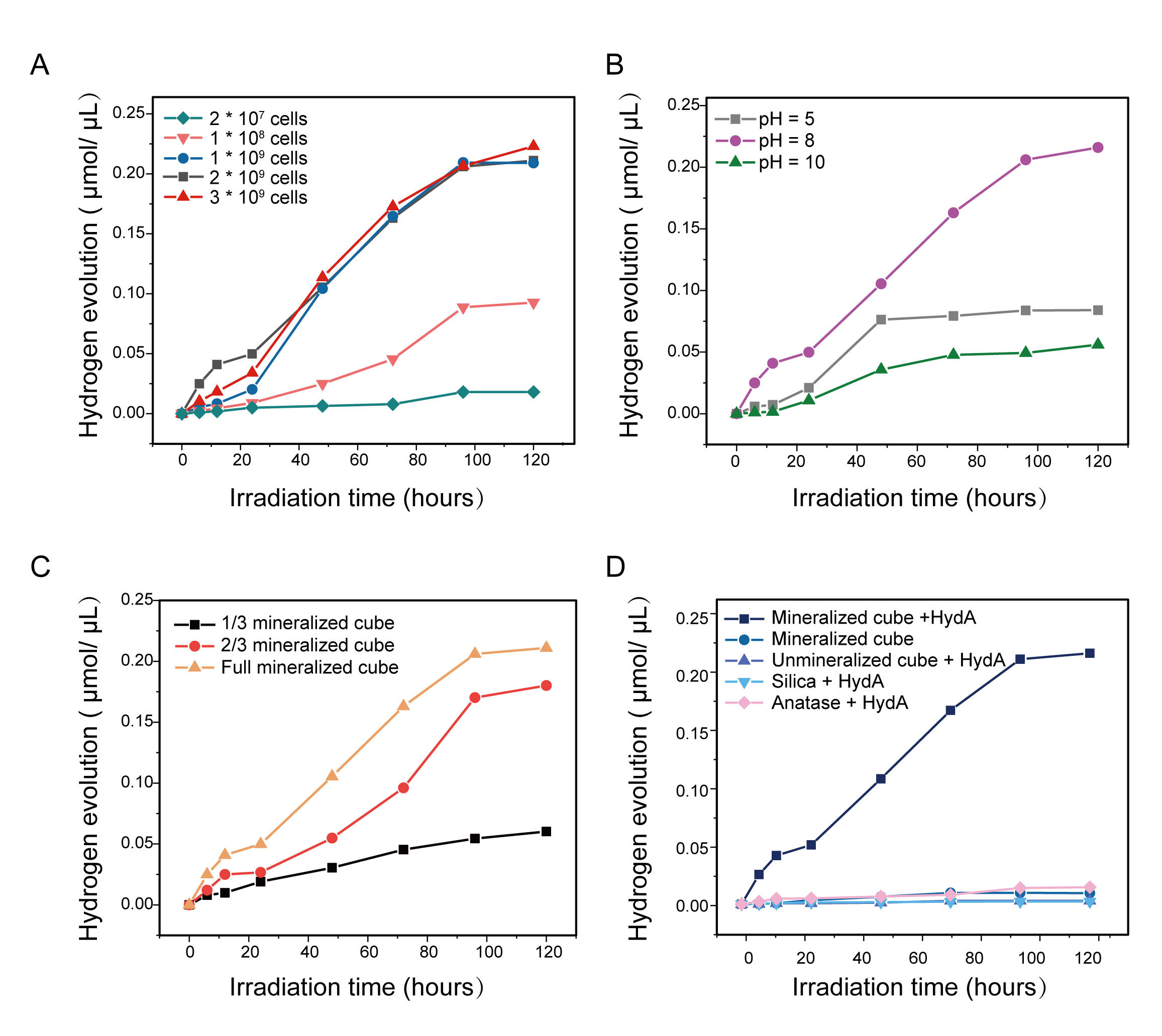
**

**Supplementary Figure 22.** Measurements of the amount of hydrogen produced by the hybrid mineralized system under different conditions. HydA, [Fe-Fe]-hydrogenase HyaABCDEF.

**Supplementary References:**

1. Bieniossek C, Nie Y, Frey D *et al.* *Nat Methods*. 2009; **6**(6): 447-50.

2. King PW, Posewitz MC, Ghirardi ML *et al.* *J Bacteriol*. 2006; **188**(6): 2163-72.

3. Velusamy P, Kumar GV, Jeyanthi V *et al.* *Toxicol Res*. 2016; **32**(2): 95-102.

4. Ruggeri FS, Adamcik J, Jeong JS *et al.* *Angew Chem Int Ed Engl*. 2015; **54**(8): 2462-6.

5. Adamcik J, Lara C, Usov I *et al.* *Nanoscale*. 2012; **4**(15): 4426-9.

6. Chowdhury S, Thomas V, Dean D *et al.* *J Nanosci Nanotechnol*. 2005; **5**(11): 1816-20.
